# Activation of the Cph1-Dependent MAP Kinase Signaling Pathway Induces White-Opaque Switching in *Candida albicans*

**DOI:** 10.1371/journal.ppat.1003696

**Published:** 2013-10-10

**Authors:** Bernardo Ramírez-Zavala, Michael Weyler, Tsvia Gildor, Christian Schmauch, Daniel Kornitzer, Robert Arkowitz, Joachim Morschhäuser

**Affiliations:** 1 Institut für Molekulare Infektionsbiologie, Universität Würzburg, Würzburg, Germany; 2 Department of Molecular Microbiology, The Rappaport Faculty of Medicine and Research Institute, Technion - I.I.T., Haifa, Israel; 3 Institute of Biology Valrose, CNRS UMR7277, INSERM UMR1091, Université Nice Sophia-Antipolis, Nice, France; Carnegie Mellon University, United States of America

## Abstract

Depending on the environmental conditions, the pathogenic yeast *Candida albicans* can undergo different developmental programs, which are controlled by dedicated transcription factors and upstream signaling pathways. *C. albicans* strains that are homozygous at the mating type locus can switch from the normal yeast form (white) to an elongated cell type (opaque), which is the mating-competent form of this fungus. Both white and opaque cells use the Ste11-Hst7-Cek1/Cek2 MAP kinase signaling pathway to react to the presence of mating pheromone. However, while opaque cells employ the transcription factor Cph1 to induce the mating response, white cells recruit a different downstream transcription factor, Tec1, to promote the formation of a biofilm that facilitates mating of opaque cells in the population. The switch from the white to the opaque cell form is itself induced by environmental signals that result in the upregulation of the transcription factor Wor1, the master regulator of white-opaque switching. To get insight into the upstream signaling pathways controlling the switch, we expressed all *C. albicans* protein kinases from a tetracycline-inducible promoter in a switching-competent strain. Screening of this library of strains showed that a hyperactive form of Ste11 lacking its N-terminal domain (Ste11^ΔN467^) efficiently stimulated white cells to switch to the opaque phase, a behavior that did not occur in response to pheromone. Ste11^ΔN467^-induced switching specifically required the downstream MAP kinase Cek1 and its target transcription factor Cph1, but not Cek2 and Tec1, and forced expression of Cph1 also promoted white-opaque switching in a Wor1-dependent manner. Therefore, depending on the activation mechanism, components of the pheromone-responsive MAP kinase pathway can be reconnected to stimulate an alternative developmental program, switching of white cells to the mating-competent opaque phase.

## Author Summary

The pathogenic yeast *Candida albicans* can switch from the white yeast form to the mating-competent opaque form. Opaque cells are less virulent than white cells, but they can avoid recognition by phagocytes, indicating that white-opaque switching has evolved as an adaptation mechanism of *C. albicans* to specific host niches. Both white and opaque cells respond to mating pheromone by activating the Ste11-Hst7-Cek1/Cek2 MAP kinase pathway, but with different outcomes. Opaque cells utilize the transcription factor Cph1 to induce the mating response, whereas white cells recruit a different downstream transcription factor, Tec1, to promote biofilm formation. We used a comprehensive protein kinase expression library to gain insight into the signaling pathways that regulate switching from the white to the opaque phase and found that a hyperactive form of the upstream kinase Ste11 induced white opaque-switching, a behavior that did not occur in response to pheromone. Hyperactive Ste11 functions *via* the opaque-specific transcription factor Cph1 instead of the white-specific transcription factor Tec1 to promote this alternative developmental program. Therefore, depending on the activation mechanism, components of the pheromone-responsive MAP kinase pathway can be rewired to stimulate a transition from the more virulent white form to the less aggressive, but mating-competent opaque form.

## Introduction

The yeast *Candida albicans* is a member of the microbiota in the gastrointestinal and genitourinary tracts of most healthy persons, but it can also cause superficial as well as life-threatening systemic infections when host defenses are compromised. Morphological transitions play a major role in the biology of *C. albicans* and in the interactions of the fungus with its host. For example, in response to various environmental stimuli, *C. albicans* alters its morphology from a unicellular budding yeast to a multicellular hyphal form. The switch from yeast to filamentous growth facilitates tissue invasion and is associated with the transition of *C. albicans* from a harmless colonizer to a pathogen that causes symptomatic infections [1].

*C. albicans* can also switch from the normal, round-to-oval yeast morphology (white) to an elongated yeast cell type (opaque), which is the mating-competent form of this diploid fungus [2]. Opaque cells can mate with opaque cells of opposite mating type to generate tetraploid fusion products, which may then undergo random chromosome loss to generate recombinant progeny in a parasexual cycle [3]. Switching of white cells to the opaque phase requires the transcription factor Wor1, the master regulator of white-opaque switching [4], [5], [6]. *WOR1* is expressed at very low levels in white cells, but an increase in the amount of Wor1 above a threshold induces switching to the opaque phase. Wor1 activates its own expression, resulting in a positive feedback loop that provides the high Wor1 levels required for maintenance of the opaque phenotype. Additional transcription factors, including the positive regulators Wor2 and Czf1 and the negative regulator Efg1, which are themselves controlled by Wor1, ensure bistable expression of *WOR1* (low in white and high in opaque cells) and epigenetic inheritance of the two phases [7]. Switching to the mating-competent opaque cell type is restricted to strains that are homozygous at the mating type locus (*MTL* **a**/**a** or *MTL*α/α). In heterozygous *MTL* **a**/α strains, switching of white cells to the opaque phase is inhibited by a heterodimeric repressor consisting of the homeodomain proteins **a**1 (encoded by *MTL* **a**) and α2 (encoded by *MTL*α), which prevents *WOR1* expression [2], [4], [5], [6]. Most *C. albicans* strains in nature are *MTL* heterozygotes, but they can become homozygous by genomic rearrangements, which relieves them from repression by the **a**1-α2 repressor and renders the cells switching-competent [8].

White and opaque cells differ not only in their mating capacity, but also in many additional phenotypes and in the expression of genes that are unrelated to mating, suggesting that they are adapted to different environments within a mammalian host [9], [10], [11]. Opaque cells colonize skin more readily than do white cells, but they are much less virulent than white cells during a systemic infection [12], [13]. Although opaque cells can form hyphae, they do not undergo the yeast-hypha transition under many conditions that stimulate hyphae formation in white cells [14], [15]. This may result in a decreased capacity of opaque cells to escape from the bloodstream and invade into tissues. On the other hand, opaque cells can avoid recognition and phagocytosis by macrophages and neutrophils under conditions in which white cells are efficiently phagocytosed [16], [17], [18]. Therefore, switching to the opaque phase not only results in the acquisition of mating competence but may also allow escape from the native immune system in certain host niches in which the more aggressive white cells will be attacked, especially after hyphae formation [19], [20].

Interestingly, both white and opaque cells respond to the presence of mating pheromone. α-pheromone, which is produced by opaque α-cells, binds to the receptor Ste2 on **a**-cells, and **a**-pheromone, produced by opaque **a**-cells, binds to the receptor Ste3 on α-cells. But while opaque cells induce a mating response that results in shmoo formation and cell fusion, pheromone binding to white cells induces the production of a biofilm, which stabilizes the pheromone gradient and facilitates mating of opaque cells [21]. This differential response of white and opaque cells is achieved by the pheromone-induced activation of a common MAP kinase cascade, consisting of the MAPKKK Ste11, the MAPKK Hst7, and the partially redundant MAPKs Cek1 and Cek2, and the cell type-specific recruitment of different downstream transcription factors, Cph1 in opaque cells and Tec1 in white cells [22], [23].

It was believed for a long time that white-to-opaque switching is a stochastic process that occurs spontaneously in few cells of a population. However, it has recently become evident that white cells can be induced under certain environmental conditions to switch *en masse* to the opaque phase [24], [25], [26], [27], [28]. Although several transcription factors have been identified that regulate white-opaque switching [4], [5], [6], [7], [28], [29], [30], little is known about the upstream signal transduction pathways that allow white cells to respond to these inducing signals. Protein kinases are common components of signaling pathways that mediate cellular reactions to external and internal signals, and the protein kinase Tpk2 has recently been implicated in the induction of switching by environmental signals [26]. In the present study, we generated a comprehensive, tetracycline-inducible protein kinase expression library to investigate whether additional kinases are involved in the control of white-opaque switching. Intriguingly, we found that the pheromone-responsive MAP kinase pathway, which promotes the mating response in opaque cells and biofilm formation in white cells, can be rewired such that white cells recruit the opaque-specific transcription factor Cph1 instead of Tec1, which then induces switching to the opaque phase.

## Results

### Identification of protein kinases that induce white-opaque switching

Artificial expression of the master regulator *WOR1*, and also of the positive regulator *CZF1*, from a constitutive or inducible promoter induces switching of white cells to the opaque phase [4], [5], [6], [7], [28], [30]. We reasoned that forced expression of protein kinases that act upstream of the transcriptional regulators to promote switching in response to environmental signals may similarly induce white cells to switch to the opaque phase. Therefore, we cloned all *C. albicans* genes encoding known or putative protein kinases and their regulators in a tetracycline-inducible gene expression cassette (see Materials and Methods). We also included 21 putative hyperactive or dominant negative alleles of these kinases in the collection. The resulting library of 160 Tet-inducible protein kinases and regulators (supplemental Table S1) was then integrated into the genome of the *C. albicans MTL*α/α strain WO-1, in which white-opaque switching was originally discovered and which has been widely used as a model strain to study this developmental program. In each case, two independent transformants were kept to confirm the reproducibility of phenotypes that were induced by the expression of a kinase.

To discover kinases whose forced expression from the Tet promoter induces switching to the opaque phase, white cells of the parental strain WO-1 and the strains containing the inducible protein kinase library were grown overnight in liquid medium in the presence or absence of doxycycline and then spread at an appropriate dilution on agar plates with or without doxycycline to allow the formation of colonies from individual cells. The results of this screening are summarized in supplemental Table S2. As expected, all strains behaved like the parental strain WO-1 when they were grown in the absence of doxycycline and showed only basal levels of spontaneous switching. In contrast, forced expression of the protein kinases *MPS1*, *RAD53*, *TPK1*, *TPK2*, and the hyperactive *STE11* ^ΔN467^ allele resulted in a strongly increased frequency of switching from the white to the opaque phase when the inducer doxycycline was present in the preculture and/or during subsequent colony growth on the agar plates. Ste11 is the MAPKKK of the pheromone-responsive MAP kinase signaling pathway (but also functions in other pathways), and in the present study we focus on the role of this pathway in the regulation of white-opaque switching in *C. albicans*. The other identified kinases will be subject of future investigations.

### The MAP kinase Cek1 and the transcription factor Cph1 mediate Ste11-induced white-opaque switching

**Figure 1:**
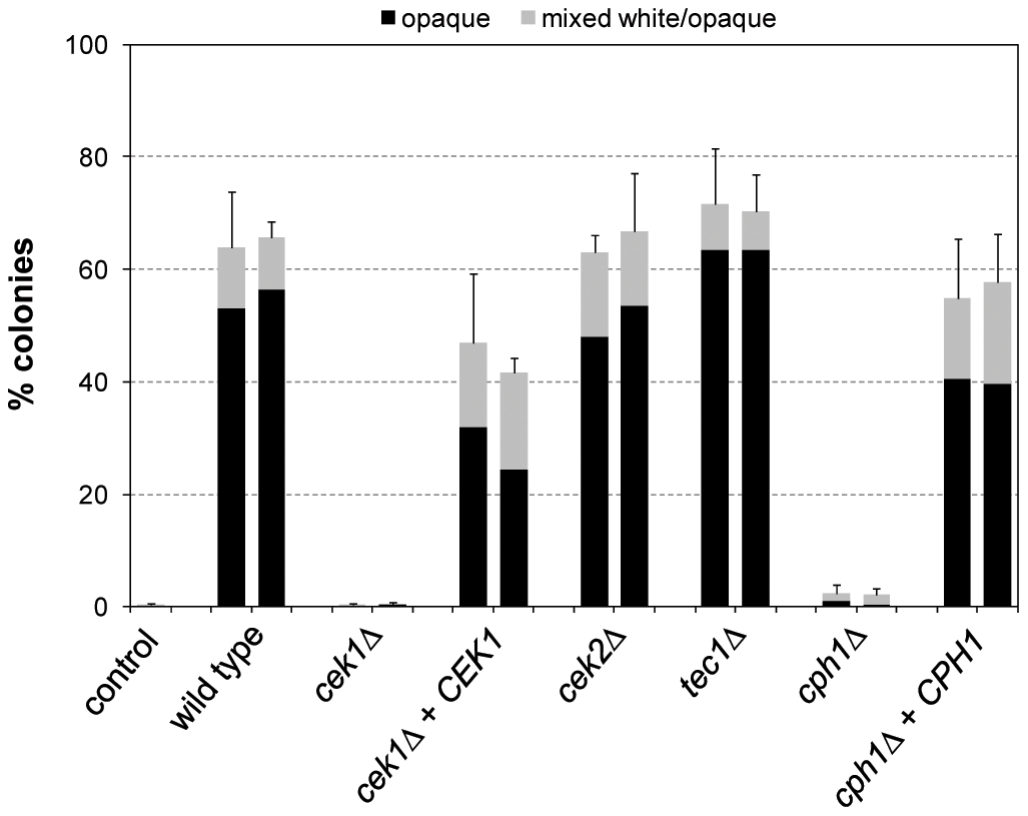
Induction of white-opaque switching by the hyperactive *STE11* ^ΔN467^ allele in the wild-type strain WO-1 and the indicated mutants. White cells of the strains were grown for 18's medium at 30°C in the presence of doxycycline to induce expression of the *STE11* ^ΔN467^ allele, diluted, and plated on Lee's agar plates without doxycycline to determine the percentage of opaque (black bars) and mixed white/opaque colonies (gray bars). Two independently constructed strains were tested in each case; the untransformed parental strain WO-1 served as control. Results are the means and standard deviations from three biological replicates. Only background switching frequencies were observed in all strains when the precultures were grown in the absence of doxycycline (not shown).

The pheromone-responsive signaling pathway contains two partially redundant MAP kinases, Cek1 and Cek2, which activate the downstream transcription factors Cph1 and Tec1 [22], [23], [31]. Of note, the functions of the two transcription factors in the pheromone response differ. While Cph1 induces the expression of mating-specific genes in opaque cells and is required for mating, Tec1 has no role in mating of opaque cells, but promotes biofilm formation of white cells in response to pheromone produced by opaque cells. This response of white cells is thought to stabilize the pheromone gradient and facilitate mating of opaque cells in a population that contains a majority of white cells [21]. We therefore investigated if these downstream MAP kinases and transcription factors are also involved in the induction of white-opaque switching by the activated Ste11^ΔN467^. To this aim, we generated deletion mutants of strain WO-1 lacking *CEK1*, *CEK2*, *CPH1*, or *TEC1* and expressed the hyperactive *STE11* ^ΔN467^ allele from the Tet promoter in the various mutants, all of which were constructed twice independently (see supplemental Table S3). As can be seen in Fig. 1, inactivation of *CEK1* abolished *STE11* ^ΔN467^-induced white-opaque switching, while deletion of *CEK2* had no effect. The switching defect of the *cek1*Δ mutants was complemented by reintroduction of a functional *CEK1* copy. These results indicate that the two MAP kinases, which have redundant functions in the mating pheromone response of opaque cells and in pheromone-induced biofilm formation of white cells [23], [31], also have divergent roles: *CEK1* is required for the Ste11-induced switching of white cells to the mating-competent opaque form, whereas *CEK2* is dispensable for this developmental program.

**Figure 2:**
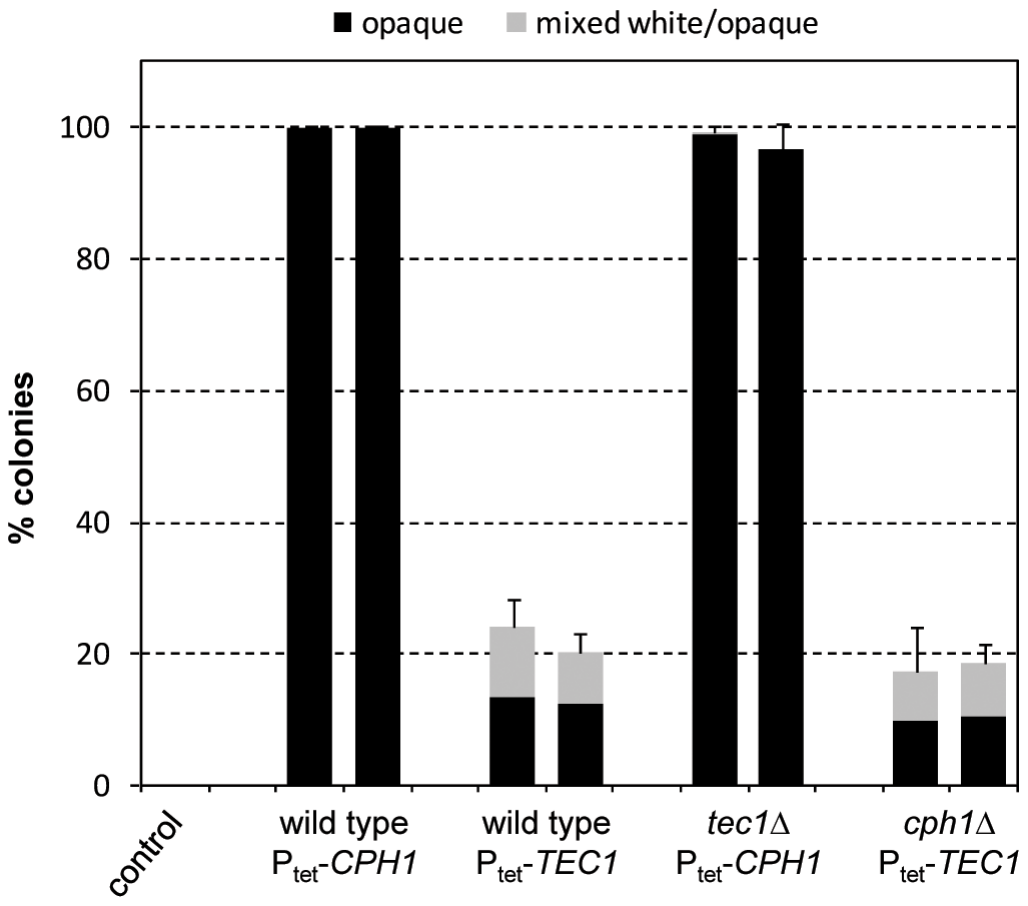
Induction of white-opaque switching by forced expression of the transcription factors *CPH1* and *TEC1* in the wild-type strain WO-1 and the indicated mutants. White cells of the strains were grown for 18's medium at 30°C in the presence of doxycycline to induce expression of *CPH1* or *TEC1*, diluted, and plated on Lee's agar plates without doxycycline to determine the percentage of opaque (black bars) and mixed white/opaque colonies (gray bars). Two independently constructed strains were tested in each case; the untransformed parental strain WO-1 served as control. Results are the means and standard deviations from three biological replicates. Only background switching frequencies were observed in all strains when the precultures were grown in the absence of doxycycline (not shown).

Deletion of *TEC1* did not affect the ability of the hyperactive Ste11 to induce white-opaque switching. In contrast, Ste11-induced switching was abolished in the *cph1*Δ mutants, and this defect was complemented by reintroduction of a functional *CPH1* copy (Fig. 1). Therefore, Cph1 not only functions in the mating response of opaque cells but is also required for Ste11-induced switching of white cells to the opaque phase. In contrast, Tec1 induces biofilm formation in white cells in response to pheromone [22], but is not required for the induction of white-opaque switching. We hypothesized that, if Cph1 is the downstream transcription factor that mediates Ste11-induced white-opaque switching, forced expression of *CPH1* might also promote switching of white cells to the opaque phase. Indeed, expression of *CPH1* from the Tet promoter in strain WO-1 strongly induced white-opaque switching (Fig. 2). Doxycycline-induced expression of *TEC1* also caused an increase in the frequency of white-opaque switching, but this was not comparable to the stimulation by *CPH1*, and *TEC1* was also not required for Cph1-induced white-opaque switching (Fig. 2). Together, these results demonstrate that activation of the pheromone-responsive MAP kinase cascade in white cells by a hyperactive form of the MAPKKK Ste11 induces one arm of this signaling pathway, including the MAPK Cek1 and the transcription factor Cph1, to promote switching to the opaque phase.

### Cph1-induced white-opaque switching requires the master regulator Wor1

**Figure 3:**
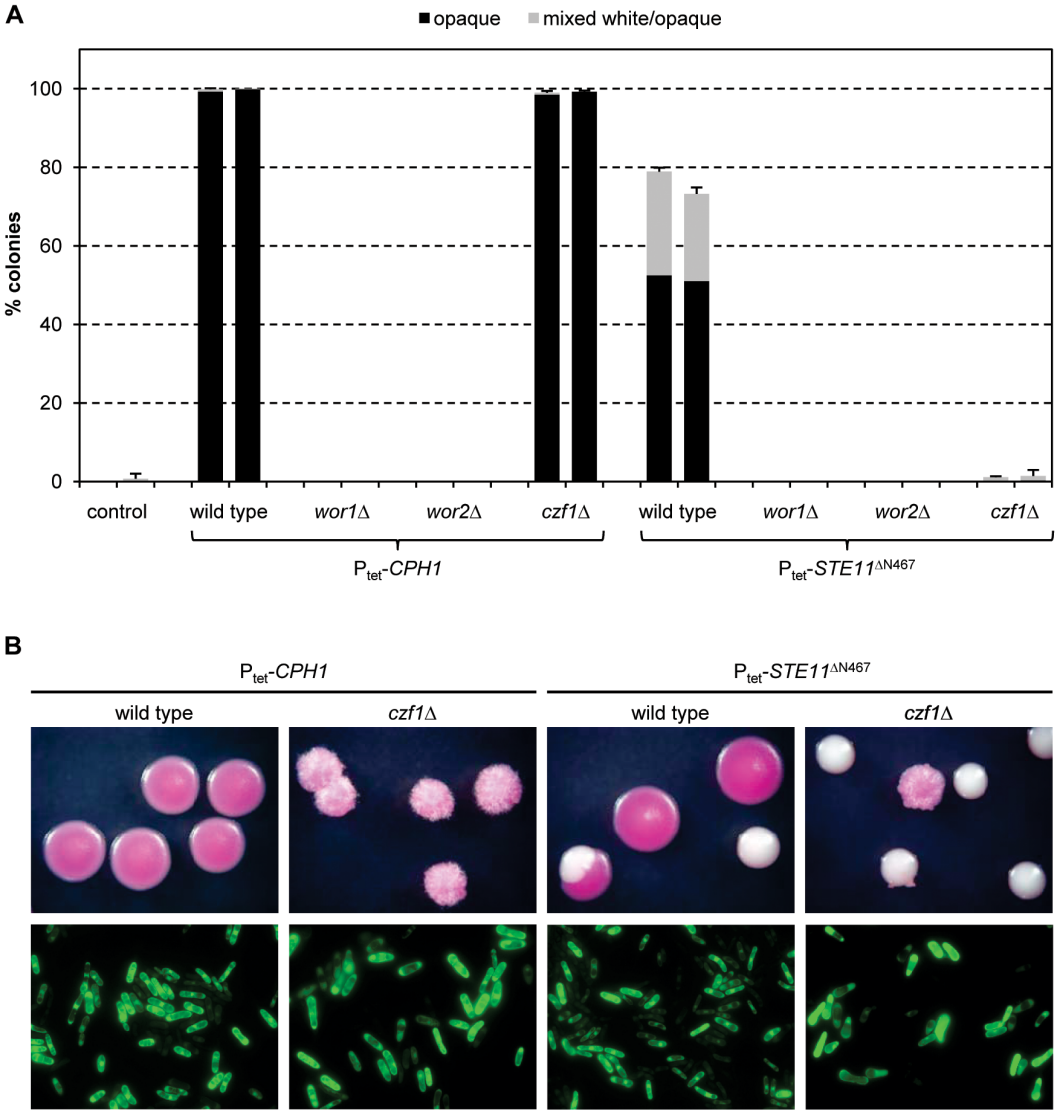
(A) Induction of white-opaque switching by forced expression of *CPH1* and *STE11* ^ΔN467^ in derivatives of the wild-type strain WO-1 and *wor1*Δ, *wor2*Δ, and *czf1*Δ mutants that express *GFP* or *RFP* from the opaque-specific *OP4* promoter. White cells of the indicated strains were grown for 18's medium at 30°C in the presence of doxycycline to induce expression of *CPH1* or *STE11* ^ΔN467^, diluted, and plated on Lee's agar plates without doxycycline to determine the percentage of opaque (black bars) and mixed white/opaque colonies (gray bars). In each case, the first columns show the results with the *GFP* reporter strains and the second columns show the results with the *RFP* reporter strains; the wild-type parental strains carrying only the reporter fusions, but not the Tet-inducible genes, served as controls. Results are the means and standard deviations from three biological replicates. Only background switching frequencies were observed in all strains when the precultures were grown in the absence of doxycycline (not shown). (B) Top panels: Colony phenotypes of opaque cells obtained after Tet-induced expression of *CPH1* or *STE11* ^ΔN467^ in wild-type and *czf1*Δ strains containing P*_OP4_-GFP* or P*_OP4_-RFP* reporter fusions. Colonies were photographed after 7 days of growth at room temperature on Lee's agar plates with phloxin B, which stains opaque colonies pink. The *GFP* and *RFP* reporter strains had identical colony phenotypes and only one of them is shown in each case; white colonies are shown for comparison. Bottom panels: Microscopic appearance of opaque cells obtained after Tet-induced expression of *CPH1* or *STE11* ^ΔN467^ in wild-type and *czf1*Δ strains containing the P*_OP4_-GFP* reporter fusion. Cells were taken from opaque colonies grown on plates without phloxin B to avoid fluorescence caused by binding of the dye. The pictures show epifluorescence micrographs of the cells with filter settings for GFP. Analogous results were obtained with the reporter strains expressing *RFP* instead of *GFP* from the *OP4* promoter (not shown).

White-opaque switching is controlled by a network of feedback loops comprising the positive regulators Wor1, Wor2, and Czf1 and the negative regulator Efg1 [7]. To investigate if the core positive regulators were required for Ste11/Cph1-induced white-opaque switching, we expressed *CPH1* and the hyperactive *STE11* ^ΔN467^ allele in mutants of strain WO-1 from which *WOR1*, *WOR2*, or *CZF1* were deleted. Initial experiments showed that the colony phenotypes of the mutants were altered in some cases, making it difficult to decide whether cells had truly switched to the opaque phase (see Fig. 3B, top panels). We therefore generated derivatives of strain WO-1 and of the mutants that expressed *GFP* or *RFP* from the opaque-specific *OP4* promoter to determine whether opaque-like colonies generated after Tet-induced expression of *CPH1* or *STE11* ^ΔN467^ contained *bona fide* opaque cells (see Fig. 3B, bottom panels). *CPH1* and *STE11* ^ΔN467^ induced white-opaque switching in the labeled wild-type reporter strains with the same efficiency as in the original parental strain WO-1 (Fig. 3A, compare with Fig. 1 and 2). No switching to the opaque phase was observed in *wor1*Δ and *wor2*Δ mutants, demonstrating that the master regulator Wor1 as well as Wor2 were also required for Cph1-induced switching. However, doxycycline-induced *CPH1* expression promoted switching to the opaque phase also in the absence of *CZF1*, while forced expression of *STE11* ^ΔN467^ failed to increase the switching frequency above background levels (Fig. 3A). Apparently, the overexpression of *CPH1* was strong enough to promote the switch in the absence of Czf1, which is part of a positive feedback loop that facilitates switching. In contrast, the comparatively weaker induction by the hyperactive *STE11* ^ΔN467^ allele was not sufficient to overcome the threshold for the switch to occur in the absence of Czf1. These results demonstrate that, when sufficiently active, Cph1 does not depend on Czf1 to promote white-opaque switching, but it still requires the master regulator Wor1.

### The hyperactive Ste11^ΔN467^ induces Cph1- and Tec1-dependent biofilm formation in white cells

**Figure 4:**
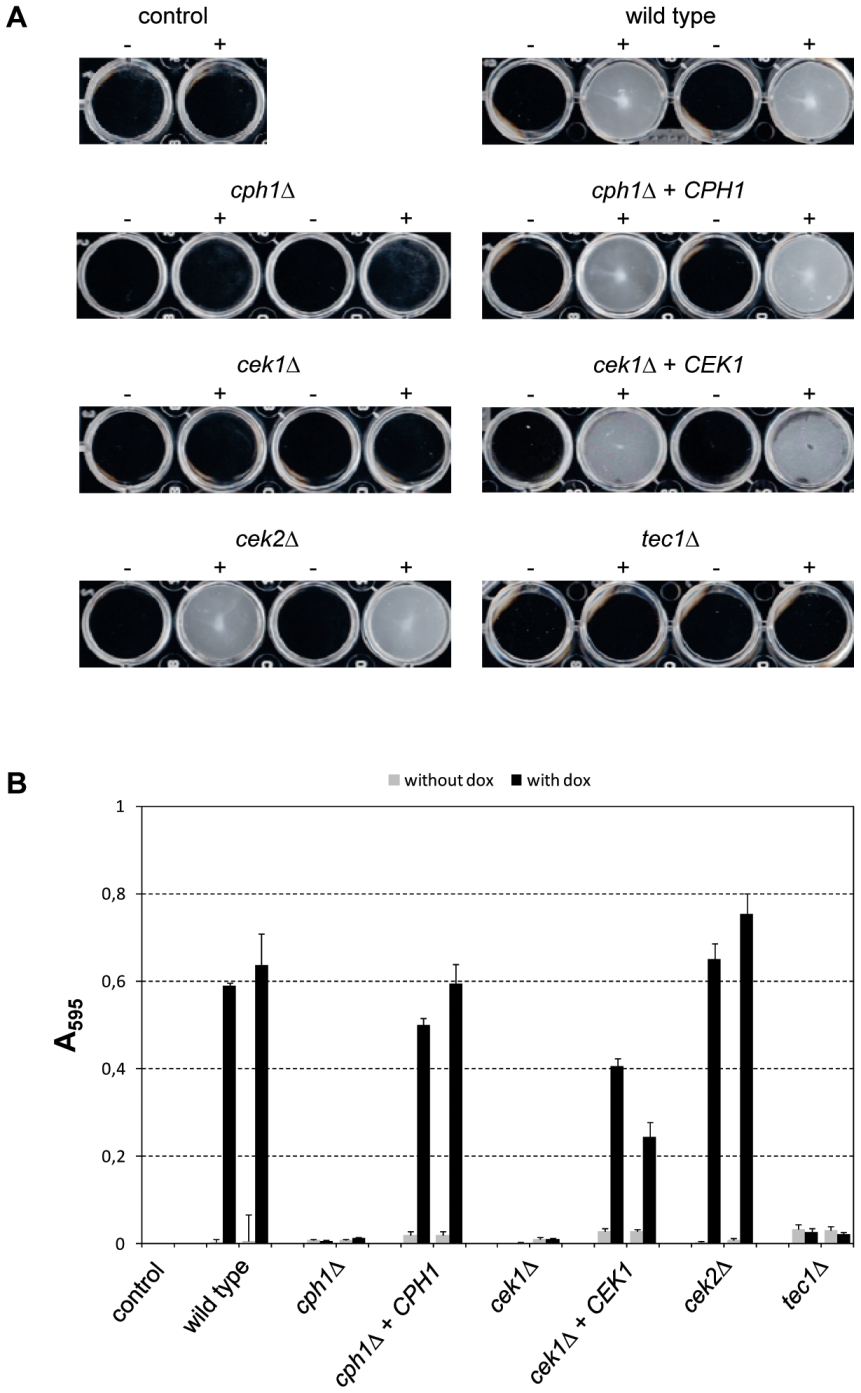
Biofilm formation by doxycycline-induced expression of the hyperactive *STE11* ^ΔN467^ allele in the wild-type strain WO-1 and the indicated mutants. White cells of the strains were grown for 24°C in liquid medium in the absence (−) or presence (+) of doxycycline. Nonadherent cells were removed by careful washing. Two independently constructed strains were tested in each case; the untransformed parental strain WO-1 served as control. (A) Photographs of the wells of the microtiter plates. (B) Quantification of biofilm formation by crystal violet staining. Results are the means and standard deviations from three biological replicates.

Tet-induced expression of *TEC1* or *STE11*, but not *CPH1*, in white cells of a *C. albicans MTL* **a**/**a** strain has been shown to promote biofilm formation under normally noninducing conditions [22]. In line with these results, we found that Tet-induced expression of the hyperactive *STE11* ^ΔN467^ allele in the *MTL*α/α strain WO-1 also induced biofilm formation in addition to white-opaque switching (Fig. 4). To investigate which downstream MAP kinases and transcription factors were required for *STE11* ^ΔN467^-induced biofilm growth, we assayed biofilm formation in *cek1*Δ, *cek2*Δ, *cph1*Δ, and *tec1*Δ mutants expressing the hyperactive *STE11* allele. As expected, *STE11* ^ΔN467^-induced biofilm formation was abolished in *tec1*Δ mutants. Surprisingly, however, the hyperactive Ste11 was also unable to promote biofilm formation in mutants lacking *CPH1*. Similarly, no induction of biofilm formation was seen in the absence of *CEK1*, while deletion of *CEK2* had no effect in these assays, demonstrating that the two MAP kinases are not redundant for Ste11^ΔN467^-induced biofilm development in strain WO-1. Reintroduction of an intact copy of *CPH1* and *CEK1* into the respective mutants restored *STE11* ^ΔN467^-induced biofilm growth, confirming that the mutant phenotype was caused by the deletion of these genes (Fig. 4).

**Figure 5:**
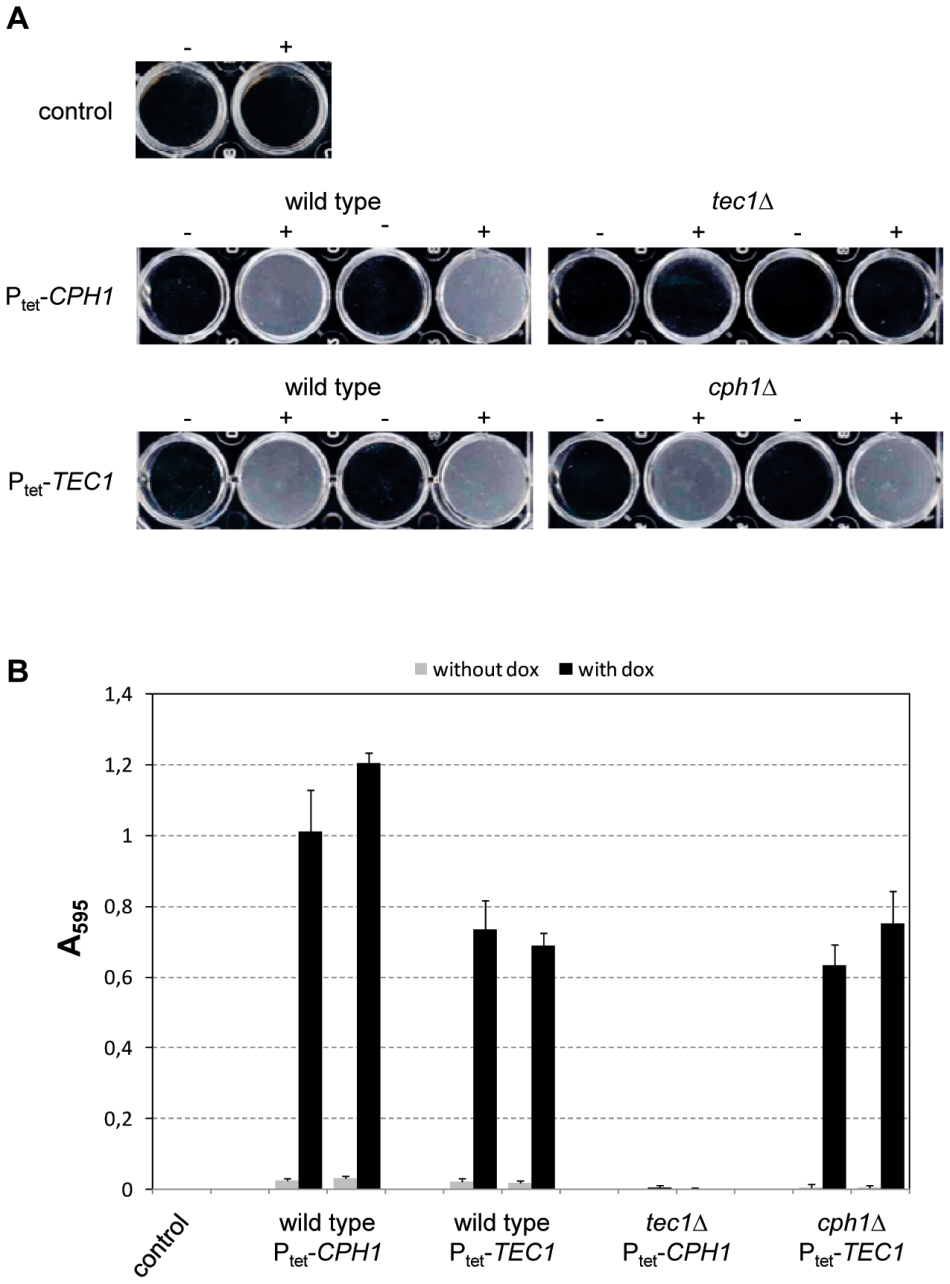
Biofilm formation by doxycycline-induced expression of *CPH1* and *TEC1* in the wild-type strain WO-1 and the indicated mutants. Strains were grown for 24°C in liquid medium in the absence (−) or presence (+) of doxycycline. Nonadherent cells were removed by careful washing. Two independently constructed strains were tested in each case; the untransformed parental strain WO-1 served as control. (A) Photographs of the wells of the microtiter plates. (B) Quantification of biofilm formation by crystal violet staining. Results are the means and standard deviations from three biological replicates.

As both *CPH1* and *TEC1* were required for the induction of biofilm formation by the hyperactive *STE11* ^ΔN467^ allele, we tested whether forced expression of either of these transcription factors would promote biofilm formation in the absence of the other in strain WO-1. Tet-induced *CPH1* expression indeed caused biofilm formation in this strain background, but this induction depended on the presence of *TEC1* (Fig. 5). In contrast, Tet-induced expression of *TEC1* promoted biofilm formation both in the presence and absence of *CPH1*.

**Figure 6:**
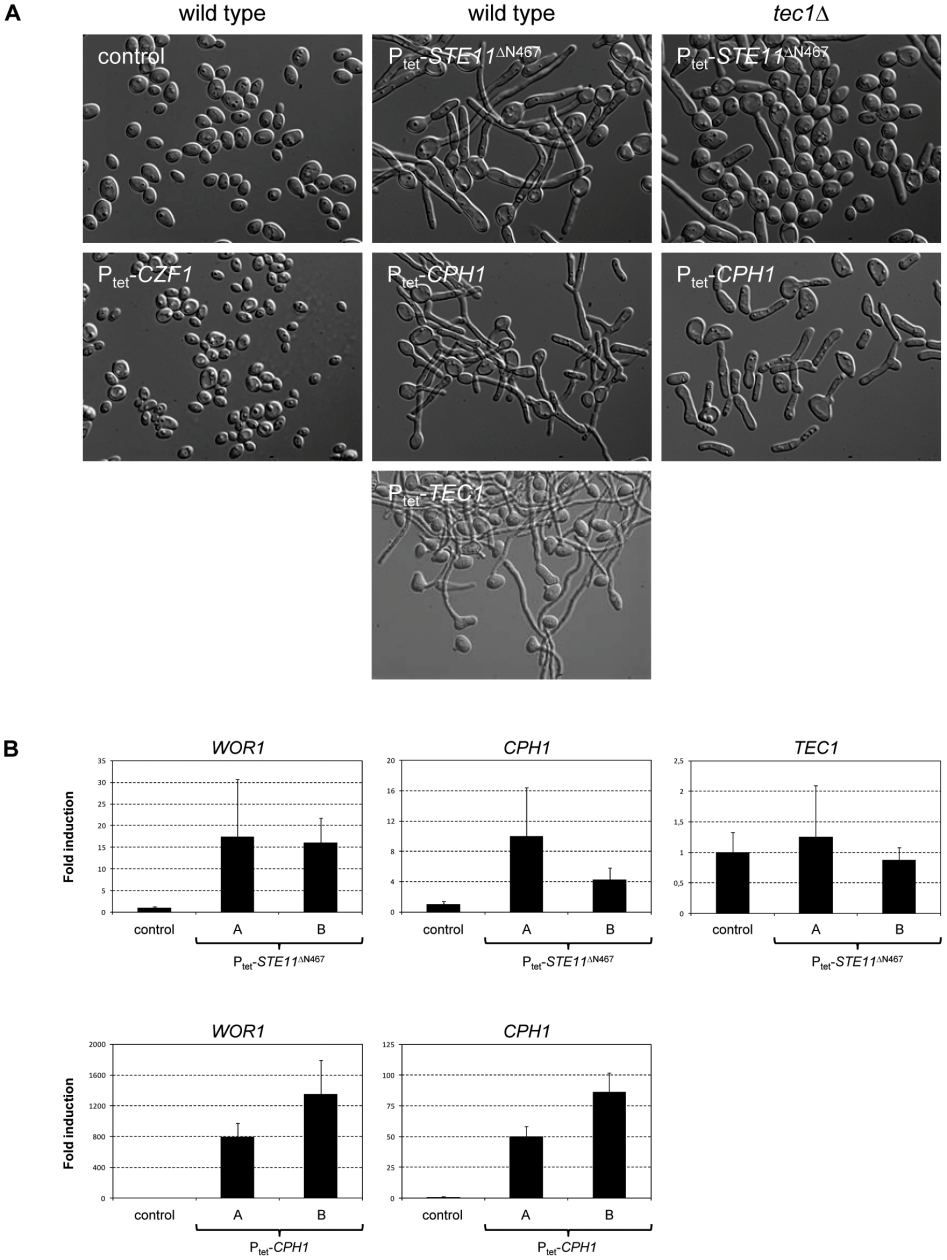
Effects of Tet-induced expression of *STE11* ^ΔN467^ and *CPH1* in wild-type and *tec1*Δ backgrounds on cellular morphology and gene expression. White cells of the strains were grown for 18(A) Microscopic appearance of the cells. The wild-type parental strain WO-1 served as control. Derivatives expressing *CZF1*, a known inducer of white-opaque switching, and *TEC1* from the Tet promoter were included for comparison. Identical results were obtained with the two independently constructed series of *STE11* ^ΔN467^ and *CPH1* expressing strains. (B) *WOR1*, *CPH1*, and *TEC1* transcript levels after Tet-induced *STE11* ^ΔN467^ or *CPH1* expression in wild-type cells were quantified by RT-qPCR. The transcript levels were compared to those in the untransformed parental strain WO-1, which were set to 1 for each gene. The results for both independently generated series of strains are shown in each case (means and standard deviations from three biological replicates). The increases in *WOR1* expression (*P* = 0.01 for P_tet_-*STE11* ^ΔN467^, *P* = 0.002 for P_tet_-*CPH1*) and *CPH1* expression (*P* = 0.04 for P_tet_-*STE11* ^ΔN467^, *P* = 0.0008 for P_tet_-*CPH1*) compared to the control were statistically significant (two-tailed Student's t-test).

The results presented above demonstrate that Tet-induced expression of the hyperactive *STE11* ^ΔN467^ allele or the downstream transcription factor *CPH1* promoted both biofilm formation and white-opaque switching in strain WO-1. Biofilm formation apparently was not a prerequisite for the induction of white-opaque switching, because switching was efficiently induced also in *tec1*Δ mutants, which did not form biofilms. Since biofilm formation has often been linked to hyphal morphogenesis, we examined the phenotype of the cells after Tet-induced *STE11* ^ΔN467^ or *CPH1* expression before plating for subsequent colony formation. For comparison, we also tested cells that expressed the known positive regulator *CZF1* from the Tet promoter. These latter cells did not yet exhibit the opaque morphology after induction of *CZF1* expression in liquid culture (Fig. 6A, bottom left panel), but were programmed to switch to the opaque phase, as almost all of them formed opaque colonies after plating (see supplemental Table S2). Wild-type cells expressing *STE11* ^ΔN467^ or *CPH1* mainly grew as filaments, similar to cells expressing *TEC1*, explaining the formation of biofilms on the plastic surface (Fig. 6A, middle panels). In the absence of *TEC1*, filamentation of cells expressing *STE11* ^ΔN467^ was strongly reduced and most cells exhibited the normal white yeast morphology (Fig. 6A, top right panel). In contrast, Tet-induced expression of *CPH1* in a *tec1*Δ background resulted in switching to the opaque phase already during growth in liquid medium, indicating that when filamentation is blocked by the absence of Tec1, upregulation of *CPH1* expression in a switching-competent strain directly promotes opaque cell formation (Fig. 6A, bottom right panel). Strikingly, many of the opaque cells formed shmoos, in accord with the previously reported finding that *CPH1* overexpression in opaque cells induces the mating response [32].

In order to understand why the filamentous cells observed after Tet-induced *STE11* ^ΔN467^ or *CPH1* expression were programmed to switch to the opaque phase after plating, we determined the expression levels of the master regulator *WOR1* in these filamentous cells by RT-qPCR. Despite some variation between biological replicates, *WOR1* transcript levels were consistently increased upon *STE11* ^ΔN467^ expression in all experiments with the two independently constructed strains, on average ca. 15-fold above those in the parental control strain (Fig. 6B, top left panel). *CPH1* transcript levels were also elevated, whereas *TEC1* mRNA levels remained unchanged (Fig. 6B, top middle and right panels). The latter result is in agreement with the fact that Cph1, but not Tec1, is the downstream transcription factor that promotes *STE11* ^ΔN467^-induced white-opaque switching. An even stronger upregulation of *WOR1* (>800-fold) was observed upon Tet-induced expression of *CPH1* itself (Fig. 6B, bottom left panel), which is explained by the significantly higher *CPH1* transcript levels in these cells compared to those seen after expression of *STE11* ^ΔN467^ from the Tet promoter (Fig. 6B, middle panels). These data demonstrate that the activation of the MAP kinase cascade in white cells by the hyperactive *STE11* ^ΔN467^ has a different outcome compared to the induction by pheromone. Instead of causing *TEC1* upregulation, Ste11 lacking its N-terminal inhibitory domain increases *CPH1* expression, which in turn induces *WOR1* expression, thereby programming the cells to switch to the opaque phase.

### Cph1-induced switching is independent of mating type

**Figure 7:**
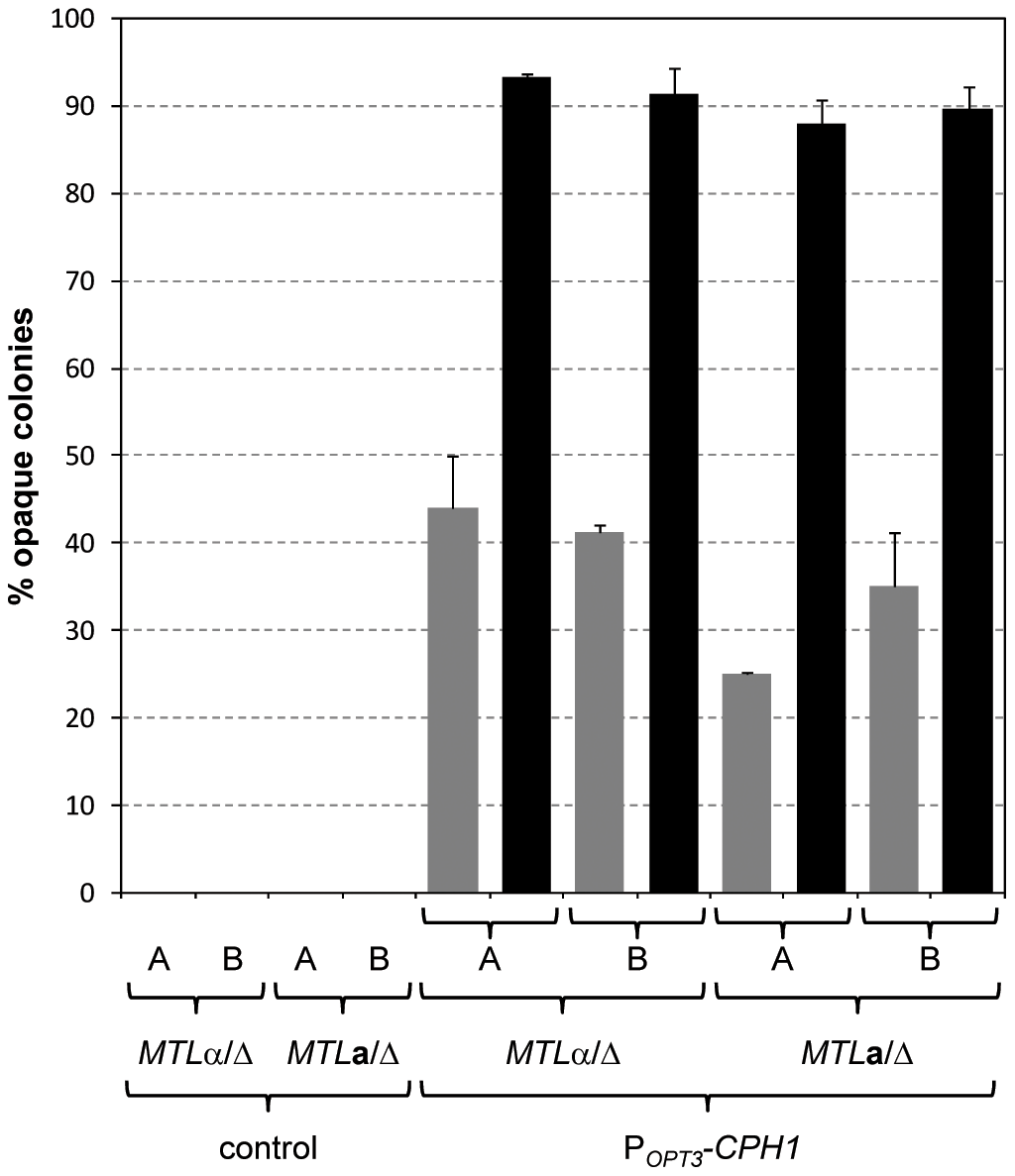
Induction of white-opaque switching in *MTL*a and *MTL*α derivatives of strain SC5314 after expression of *CPH1* from the inducible *OPT3* promoter. White cells of the strains were grown for 18-BSA-YE medium at 30°C, diluted, and plated on Lee's medium with phloxin B to determine the percentage of opaque colonies (including a small number of mixed white/opaque colonies). Two independently constructed series (A and B) of *MTL* **a** and *MTL*α strains were tested in each case; the untransformed parental strains served as controls. The gray and black bars show the results with transformants in which the P*_OPT3_-CPH1* fusion was integrated into the *OPT3-2* and *OPT3-1* allele, respectively, which could be distinguished by a SpeI restricton site polymorphism [33]. Results are the means and standard deviations from three biological replicates. Only background switching frequencies were observed when the strains were grown in noninducing YPD medium (not shown).

White-opaque switching can be induced by different environmental signals, but there are strain-specific differences in the response of switching-competent strains to the various stimuli [24], [25], [26], [28]. In strain WO-1, switching is strongly induced by a transient incubation in an anaerobic environment (0% O_2_, 18% CO_2_), while other tested strains did not switch to the opaque phase under these conditions [28]. We therefore investigated if Cph1-induced white-opaque switching might be a peculiar characteristic of strain WO-1 or if Cph1 can promote switching also in other *C. albicans* strains and in both mating types. For this purpose, we deleted either the *MTL* **a** or the *MTL*α locus in the commonly used reference strain SC5314 to generate switching-competent α and **a** derivatives, respectively, into which the P_tet_-*CPH1* fusion was subsequently introduced. Doxycycline did not induce white-opaque switching in these strains; however, using a control P_tet_-*GFP* reporter fusion we found that the Tet promoter was much less efficiently induced in this strain background than in strain WO-1 (unpublished results). Hence, we expressed *CPH1* from another regulatable promoter, P*_OPT3_*, which is efficiently induced in strain SC5314 when the cells grow on BSA as a nitrogen source [33]. Expression of *CPH1* from the *OPT3* promoter during growth in YCB-BSA-YE medium strongly stimulated white-opaque switching in independently generated **a** and α derivatives of strain SC5314 (Fig. 7). Incubation of the untransformed parental strains in the same growth medium did not promote white-opaque switching, confirming that switching was induced by *CPH1* expression. We consistently observed a higher switching frequency when the P*_OPT3_*-*CPH1* fusion was integrated into the *OPT3-1* allele as compared to the *OPT3-2* allele. Allele-specific differences in the activity of the *OPT3* promoter did not seem to be the reason, because a P*_OPT3_-GFP* reporter fusion was expressed at comparable levels after integration at either of the two loci (data not shown). Therefore, minor differences in the resulting *OPT3-CPH1* hybrid transcripts (e.g., stability or translational efficiency) may specifically affect Cph1 levels and, consequently, the switching frequency. Regardless, these results demonstrate that Cph1-induced white-opaque switching is not specific to strain WO-1 and is independent of mating type.

### Analysis of the information flow through the MAP kinase pathway

#### Activation of the MAP kinases Cek1 and Cek2

**Figure 8:**
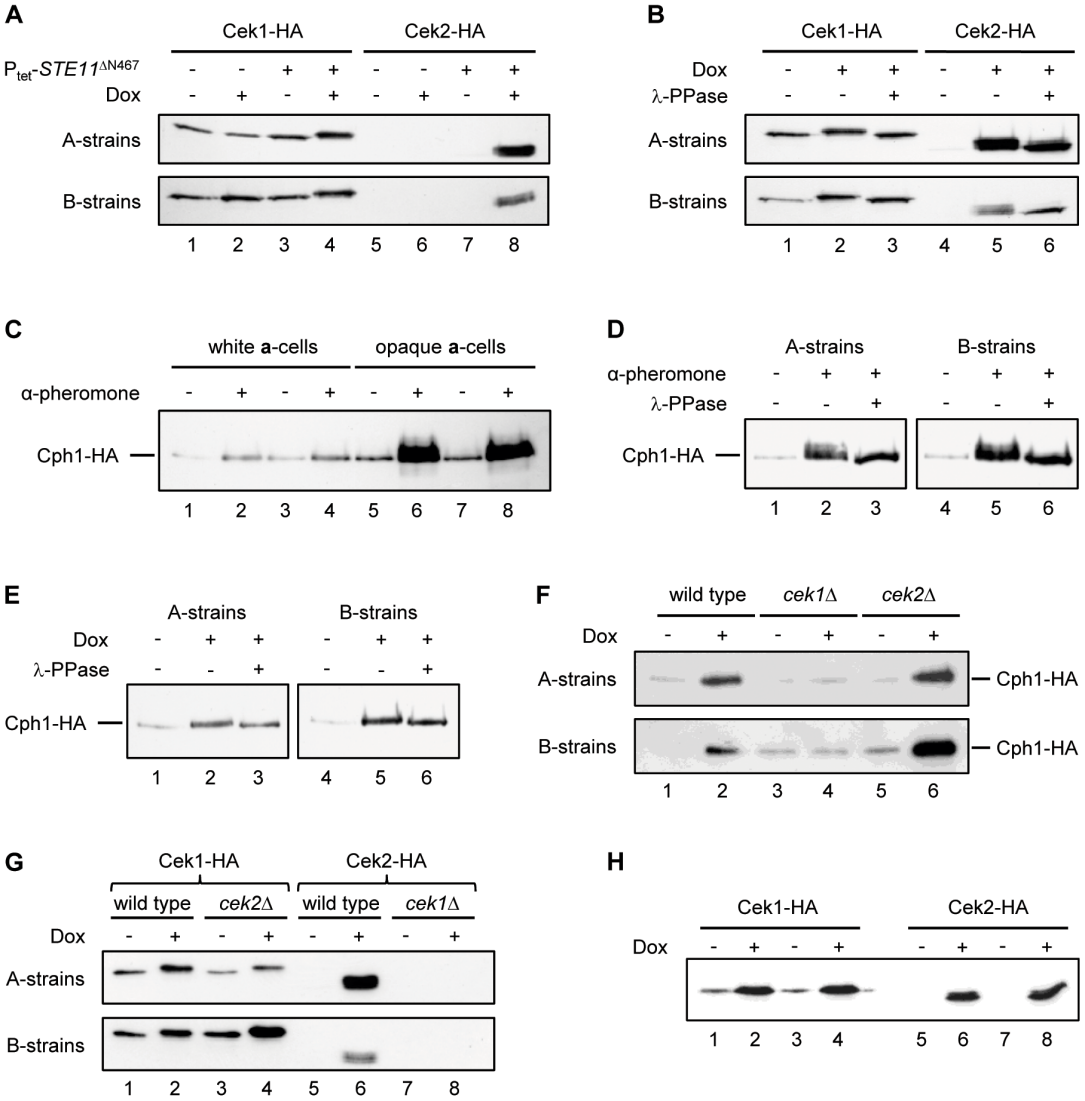
Detection of epitope-tagged Cek1, Cek2, and Cph1 in *C. albicans* cells by western immunoblotting with an anti-HA antibody. Results for two independently constructed series of strains are shown in each case. (A) White cells of strain WO-1 containing HA-tagged *CEK1* or *CEK2* alleles in the absence (−) or presence (+) of the Tet-inducible *STE11* ^ΔN467^ were grown for 18 h in liquid Lee's medium at 30°C without or with doxycycline as indicated. (B) Cell extracts of the strains containing the Tet-inducible *STE11* ^ΔN467^ were analyzed with and without phosphatase treatment as indicated. (C) White and opaque cells of *MTL* **a** derivatives of strain SC5314 containing an HA-tagged *CPH1* allele were grown for 18 h in liquid Lee's medium at 30°C in the presence or absence of α-pheromone. (D) Opaque cell extracts were analyzed with and without phosphatase treatment as indicated. (E) White cells of strain WO-1 containing a single, HA-tagged *CPH1* allele and the Tet-inducible *STE11* ^ΔN467^ were grown for 18 h in liquid Lee's medium at 30°C without or with doxycycline. Whole cell extracts were analyzed with or without prior phosphatase treatment as indicated. (F) Cph1 upregulation by the hyperactive Ste11 depends on Cek1, but not Cek2. White cells containing an HA-tagged *CPH1* allele and the Tet-inducible *STE11* ^ΔN467^ in the indicated genetic backgrounds were grown for 18 h in liquid Lee's medium at 30°C without or with doxycycline as indicated. (G) Cek2 upregulation by the hyperactive Ste11 depends on Cek1. White cells containing HA-tagged *CEK1* or *CEK2* alleles and the Tet-inducible *STE11* ^ΔN467^ in the indicated genetic backgrounds were grown for 18 h in liquid Lee's medium at 30°C without or with doxycycline as indicated. (H) Overexpression of *CPH1* results in increased levels of Cek1 and Cek2. White cells containing HA-tagged *CEK1* or *CEK2* alleles and a Tet-inducible *CPH1* were grown for 18 h in liquid Lee's medium at 30°C without or with doxycycline as indicated.

To understand in more detail how activation of the MAP kinase pathway results in Cph1-mediated white-opaque switching, we first investigated the effect of the hyperactive Ste11 on the downstream MAP kinases Cek1 and Cek2. For this purpose, we introduced the Tet-inducible *STE11* ^ΔN467^ allele into derivatives of strain WO-1 in which one of the two alleles of *CEK1* or *CEK2* was labeled with a 3×HA tag. The tagged Cek1 was readily detectable by western immunoblot analysis in the uninduced strains and in control strains without the *STE11* ^ΔN467^ allele (Fig. 8A, lanes 1–3 and Fig. 8B, lane 1), whereas Cek2 was hardly visible (Fig. 8A, lanes 5–7 and Fig. 8B, lane 4). Doxycycline-induced expression of the *STE11* ^ΔN467^ allele resulted in reduced mobility of Cek1 (compare lanes 3 and 4 in Fig. 8A and lanes 1 and 2 in Fig. 8B). This shift in mobility was abolished when the cell extracts were treated with λ-phosphatase (Fig. 8B, lane 3), indicating that Cek1 was phosphorylated in the presence of the hyperactive Ste11. Interestingly, expression of *STE11* ^ΔN467^ also caused strongly increased Cek2 levels (Fig. 8A, lane 8 and Fig. 8B, lane 5). Cek2 was detected as two to three bands, and the bands with lower mobility disappeared after phosphatase treatment, indicating that Cek2 was also phosphorylated, but less efficiently than Cek1.

#### Activation of the transcription factor Cph1

As shown in Fig. 6B, the hyperactive Ste11 caused upregulation of the downstream transcription factor *CPH1*, which in turn stimulated white-opaque switching. Other researchers have also observed increased expression of *CPH1* upon pheromone treatment of opaque cells [23], [34]. As the downstream target of a MAP kinase pathway, Cph1 is likely to be activated by phosphorylation, but Cph1 phosphorylation has not been investigated so far. To test whether a possible phosphorylation of Cph1 would be detectable by a change in protein mobility, we labeled one of the *CPH1* alleles in *MTL*a derivatives of strain SC5314 with a 3×HA tag. When opaque cells of these strains were treated with α-pheromone, the levels of Cph1 increased substantially, in line with the previously reported upregulation of *CPH1* expression by pheromone (Fig. 8C, lanes 5–8). Shorter exposure of the blots revealed a new Cph1 band with slower mobility, which disappeared after phosphatase treatment, indicating that it is a phosphorylated form of Cph1 (Fig. 8D). When white cells of the same strains were treated with α-pheromone, only a slight increase in Cph1 levels was observed (Fig. 8C, lanes 1–4). We then tested whether expression of the hyperactive *STE11* ^ΔN467^ would also result in Cph1 phosphorylation in white cells. To this aim, we expressed the Tet-inducible *STE11* ^ΔN467^ in derivatives of strain WO-1 carrying a single, HA-tagged *CPH1* allele. Expression of the hyperactive *STE11* resulted in increased Cph1 protein levels, but no band corresponding to the phosphorylated form of Cph1 in pheromone-treated opaque cells was detected (Fig. 8E). However, this result does not exclude the existence of a different phosphorylated form of Cph1 that did not exhibit an altered mobility. Of note, the increase in Cph1 levels caused by the hyperactive Ste11 was comparable to that seen in opaque cells upon pheromone treatment (compare Fig. 8D and E). Therefore, Ste11-induced white-opaque switching was not caused by unphysiologically high amounts of Cph1. We then compared the levels of Cph1 in the wild type and in strains lacking Cek1 or Cek2. For this purpose, one of the resident *CPH1* alleles was labeled with the 3×HA tag in the parental strain WO-1 and the *cek1*Δ and *cek2*Δ mutants. Fig. 8F shows that the upregulation of Cph1 by the hyperactive Ste11 did not depend on Cek2, but was abolished in *cek1*Δ mutants, in line with the Cek1-dependent induction of switching.

To understand why Ste11-induced upregulation of Cph1 in white cells and switching to the opaque phase depended on Cek1, but not Cek2, although both MAP kinases became activated upon expression of the hyperactive *STE11* ^ΔN467^ allele, we monitored the levels of HA-tagged Cek1 and Cek2 in the absence of the other MAP kinase. While Cek1 was activated also in *cek2*Δ mutants, the upregulation of Cek2 observed in a wild-type background was abolished in *cek1*Δ mutants (Fig. 8G). We reasoned that Cek1-mediated activation of Cph1 was responsible for the upregulation of Cek2. To test this hypothesis, we expressed *CPH1* from the Tet promoter in strains containing HA-tagged *CEK1* or *CEK2*. Fig. 8H shows that Tet-induced expression of *CPH1* resulted in strongly increased levels of both Cek1 and Cek2. Therefore, activation of Cph1 by the upstream kinase Cek1 further stimulates the pathway by a positive feedback mechanism that provides higher amounts of both MAP kinases.

**Figure 9:**
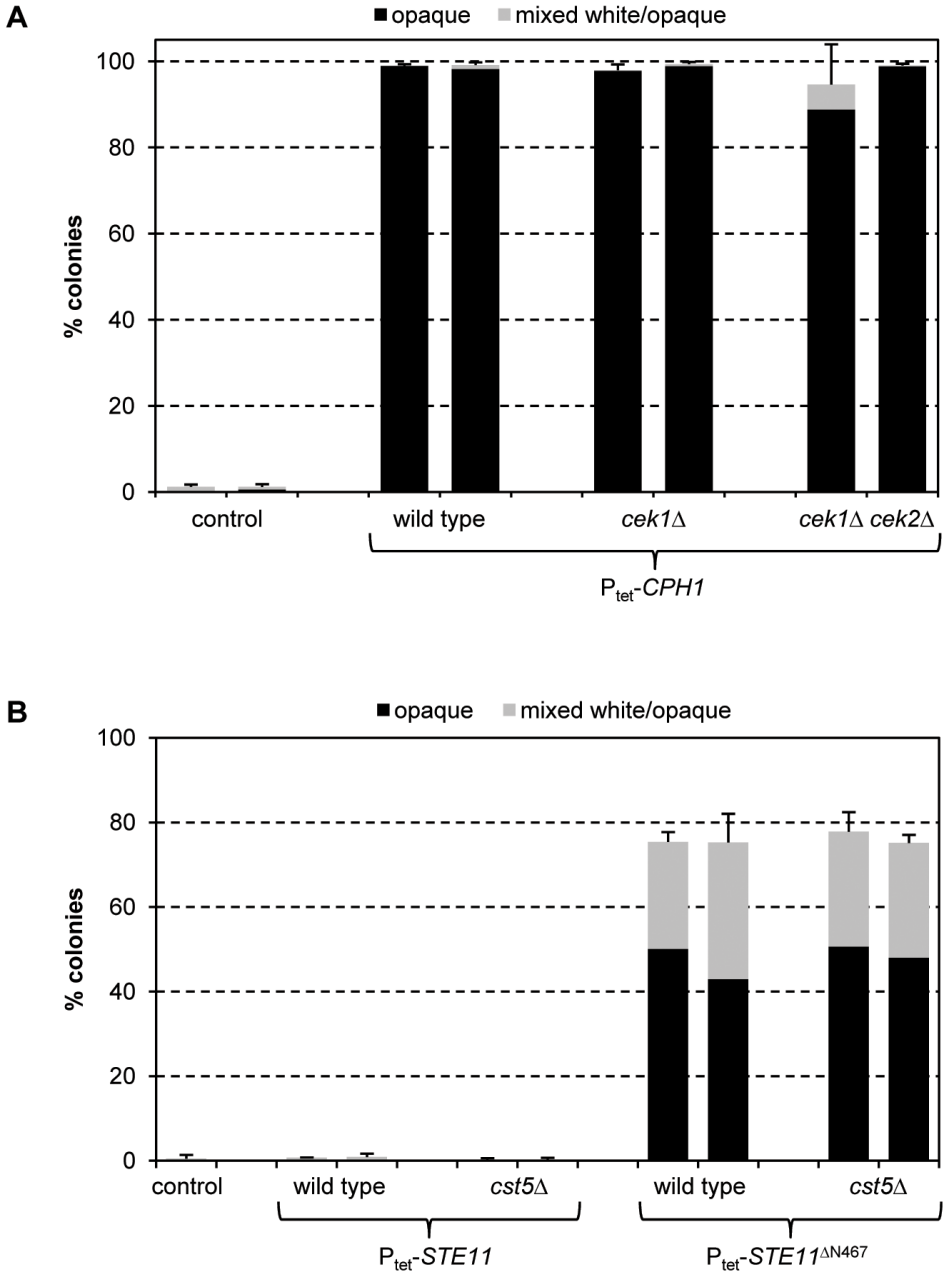
(A) Overexpression of *CPH1* induces white-opaque switching independently of the upstream MAP kinases Cek1 and Cek2. White cells of strains containing the Tet-inducible *STE11* ^ΔN467^ allele in the indicated backgrounds were grown for 18 h in liquid Lee's medium at 30°C in the presence of doxycycline, diluted, and plated on Lee's agar plates without doxycycline to determine the percentage of opaque (black bars) and mixed white/opaque colonies (gray bars). Two independently constructed strains were tested in each case; the untransformed parental strain WO-1 served as control in two sets of experiments. Results are the means and standard deviations from three (*cek1*Δ and *cek1*Δ *cek2*Δ mutants) or six (wild-type strains) biological replicates. Only background switching frequencies were observed in all strains when the precultures were grown in the absence of doxycycline (not shown). (B) The scaffold protein Cst5 is not required for the induction of white-opaque switching by the hyperactive Ste11^ΔN467^. White cells of strains containing a Tet-inducible wild-type *STE11* or *STE11* ^ΔN467^ in wild-type or *cst5*Δ backgrounds were grown for 18 h in liquid Lee's medium at 30°C in the presence of doxycycline, diluted, and plated on Lee's agar plates without doxycycline to determine the percentage of opaque (black bars) and mixed white/opaque colonies (gray bars). Two independently constructed strains were tested in each case; the untransformed parental strain WO-1 served as control. Results are the means and standard deviations from three biological replicates. Only background switching frequencies were observed in all strains when the precultures were grown in the absence of doxycycline (not shown).

To investigate whether overexpression of *CPH1* in white cells was sufficient to induce switching to the opaque phase or if phosphorylation by the upstream MAP kinases was required, we expressed the Tet-inducible *CPH1* in mutants lacking *CEK1* or both *CEK1* and *CEK2*. As can be seen in Fig. 9A, doxycycline-induced *CPH1* expression efficiently stimulated switching even in the absence of both upstream MAP kinases. Therefore, overexpression of *CPH1* is sufficient for its ability to induce white-opaque switching, indicating that alternative pathways that upregulate *CPH1* expression may also promote switching under appropriate conditions.

#### Role of the scaffold protein Cst5

The scaffold protein Cst5 is required for both the Cph1-mediated mating response of opaque cells and Tec1-dependent biofilm formation of white cells [32]. As the hyperactive Ste11^ΔN467^ lacks the binding site for Cst5 [35], we investigated whether it could promote white-opaque switching independently of the scaffold protein. Indeed, Tet-induced expression of *STE11* ^ΔN467^ stimulated switching in *cst5*Δ mutants of strain WO-1 with the same efficiency as in the parental wild-type strain (Fig. 9B). This result suggested that the hyperactive Ste11 activated the downstream components of the pathway in a manner that is different from that observed in response to pheromone. Tet-induced expression of wild-type *STE11* did not promote switching in the presence or absence of *CST5*, indicating that the mere absence of the scaffold protein was not sufficient and additional signals are required to change the output of Ste11 activation in white cells.

### Environmental signals can induce white-opaque switching in the absence of Cph1

**Figure 10:**
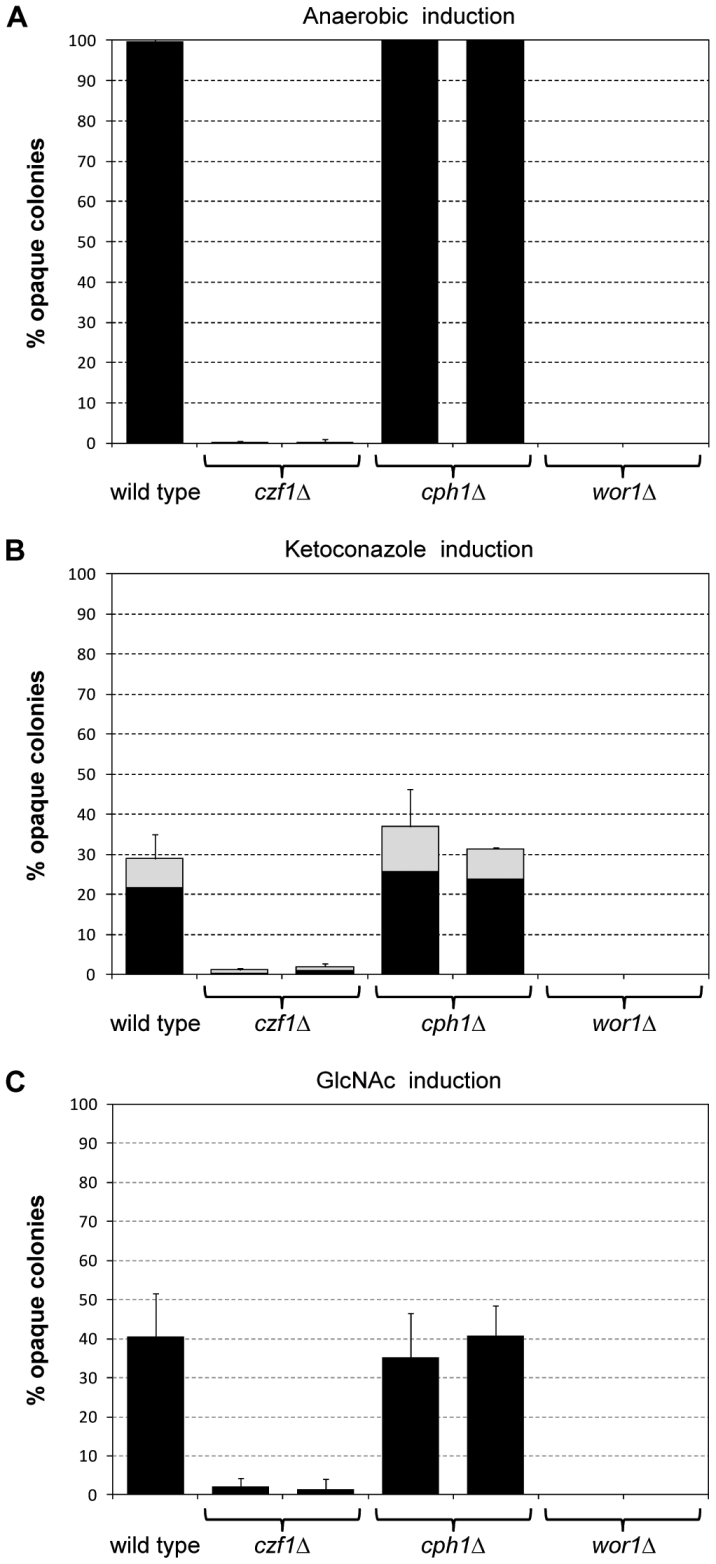
Induction of white-opaque switching by different environmental stimuli in strain WO-1 and *czf1*Δ, *cph1*Δ and *wor1*Δ mutants. Two independently generated series of mutants were analyzed in each case and the percentage of opaque (black bars) and mixed white/opaque colonies (gray bars) was determined (means and standard deviations from three biological replicates). (A) Induction by anaerobic conditions. White cells from a YPD preculture were diluted 10^−5^, plated on Lee's medium, and incubated for 2 days at room temperature in an anaerobic jar, followed by incubation under aerobic conditions at room temperature for 7 further days to allow colony development. (B) Induction by ketoconazole. White cells from a YPD preculture were diluted 10^−2^ in YPD medium containing 50 µM ketoconazole and incubated for 1 day at 30°C without shaking. Appropriate dilutions were then spread on Lee's plates and incubated for 7 days at room temperature. Control cells incubated in the presence of solvent (DMSO) alone showed <3% opaque or mixed white/opaque colonies. (C) Induction by GlcNAc. White cells were grown for 2 days in YPD medium, diluted 10^−5^, plated on Lee's medium with 1.25% GlcNAc instead of glucose, and incubated for 7 days at room temperature.

The finding that activation of the Cph1-dependent MAP kinase pathway in white cells promoted switching to the opaque phase suggested that the presence of mating pheromone might also stimulate white-opaque switching. However, in contrast to opaque cells, white cells do not [22], [23] or not strongly (Fig. 8C) upregulate *CPH1* expression in response to pheromone and no pheromone-induced white-opaque switching has been reported so far. In line with this, the addition of synthetic α-pheromone to white **a**-cells derived from strain SC5314 did not induce switching to the opaque phase under various growth conditions tested, and a mixture of opaque **a**- and α-derivatives of strain SC5314 (used as a source of **a**-pheromone) also did not stimulate white-opaque switching in the *MTL*α/α strain WO-1 when the cells were coincubated (data not shown, see Materials and Methods for details). As white cells use Tec1 to induce biofilm formation in response to pheromone, we also tested the effect of **a**-pheromone on *tec1*Δ mutants of strain WO-1; however, no pheromone-induced switching was observed in the mutants. Consequently, other signals may activate the MAP kinase pathway in a different way from that stimulated by pheromone to result in *CPH1* instead of *TEC1* upregulation in white cells, similar to the effect of the hyperactive Ste11^ΔN467^. We therefore investigated whether Cph1 is required for white-opaque switching in response to signals that efficiently stimulate switching in strain WO-1. For this purpose, we determined the switching frequency of the wild-type strain WO-1 and the *cph1*Δ mutants under various inducing conditions; mutants lacking the master regulator Wor1 or the positive regulator Czf1 were included for comparison. In contrast to *czf1*Δ mutants, in which the frequency of switching was drastically reduced under all tested conditions (no switching was observed in *wor1*Δ mutants, as expected), cells lacking Cph1 switched to the opaque phase with the same efficiency as the parental wild-type strain WO-1 when switching was induced by incubation in an anaerobic jar, in the presence of ketoconazole, or by growth on GlcNAc as carbon source (Fig. 10). Therefore, conditions that induce white cells to switch to the opaque phase by activating *CPH1* remain to be discovered (see discussion).

## Discussion

The recent observation by several groups that switching of white cells to the opaque phase does not only occur stochastically in few cells of a population, but can be strongly stimulated by certain environmental cues [24], [25], [26], [28], suggested that expression of the master regulator *WOR1* is induced by upstream signal transduction pathways in response to these signals. The cAMP/PKA signaling pathway has been implicated in the environmental induction of white-opaque switching, because deletion of components of this pathway reduced, albeit did not abolish, the stimulation of switching by GlcNAc and elevated CO_2_ concentrations [25], [26]. In this work, we used an overexpression approach to identify protein kinases that stimulate white-opaque switching. By generating and screening a comprehensive library of *C. albicans* strains that express all protein kinases of this organism from a tetracycline-inducible promoter, we discovered several kinases that could induce white cells to switch to the opaque phase. In addition to the recently identified Tpk2 [26], we found that the homologous kinase Tpk1 as well as the kinases Mps1 and Rad53 also stimulated white-opaque switching when overexpressed from the Tet promoter. The involvement of different protein kinases in the regulation of white-opaque switching probably reflects the fact that a variety of signals stimulate white cells to switch to the opaque phase. It should be noted that additional kinases not identified in our screening could nevertheless be involved in the control of white-opaque switching, because overexpression of a wild-type kinase may not necessarily have a phenotypic effect (as was the case for wild-type *STE11*, which did not stimulate switching).

A particularly intriguing result of our present study was that a hyperactive form of the MAPKKK Ste11, which acts in the pheromone response pathway, also promoted switching. This finding came as a surprise, because the pheromone-responsive MAP kinase signaling pathway is known to induce the mating response in opaque cells and biofilm formation in white cells, but so far it has not been implicated in the regulation of the switching event itself. No induction of white-opaque switching by pheromone has been reported in previous studies investigating the pheromone response of white cells [21], [23], and we also did not observe stimulation of switching by pheromone, suggesting that the MAP kinase pathway can be activated in an alternative way in white cells to promote switching to the opaque phase. The other kinases identified in our study do not seem to function *via* the MAP kinase pathway, because Tet-induced expression of *MPS1*, *RAD53*, *TPK1*, and *TPK2* stimulated switching also in *cph1*Δ and *ste11*Δ mutants (see supplemental Fig. S1), arguing that these kinases function *via* alternative signaling pathways to activate *WOR1*. Unlike the hyperactive *STE11*, these other kinases also did not promote biofilm formation in our assays (data not shown), supporting the assumption that they do not activate the MAP kinase pathway under these conditions.

**Figure 11:**
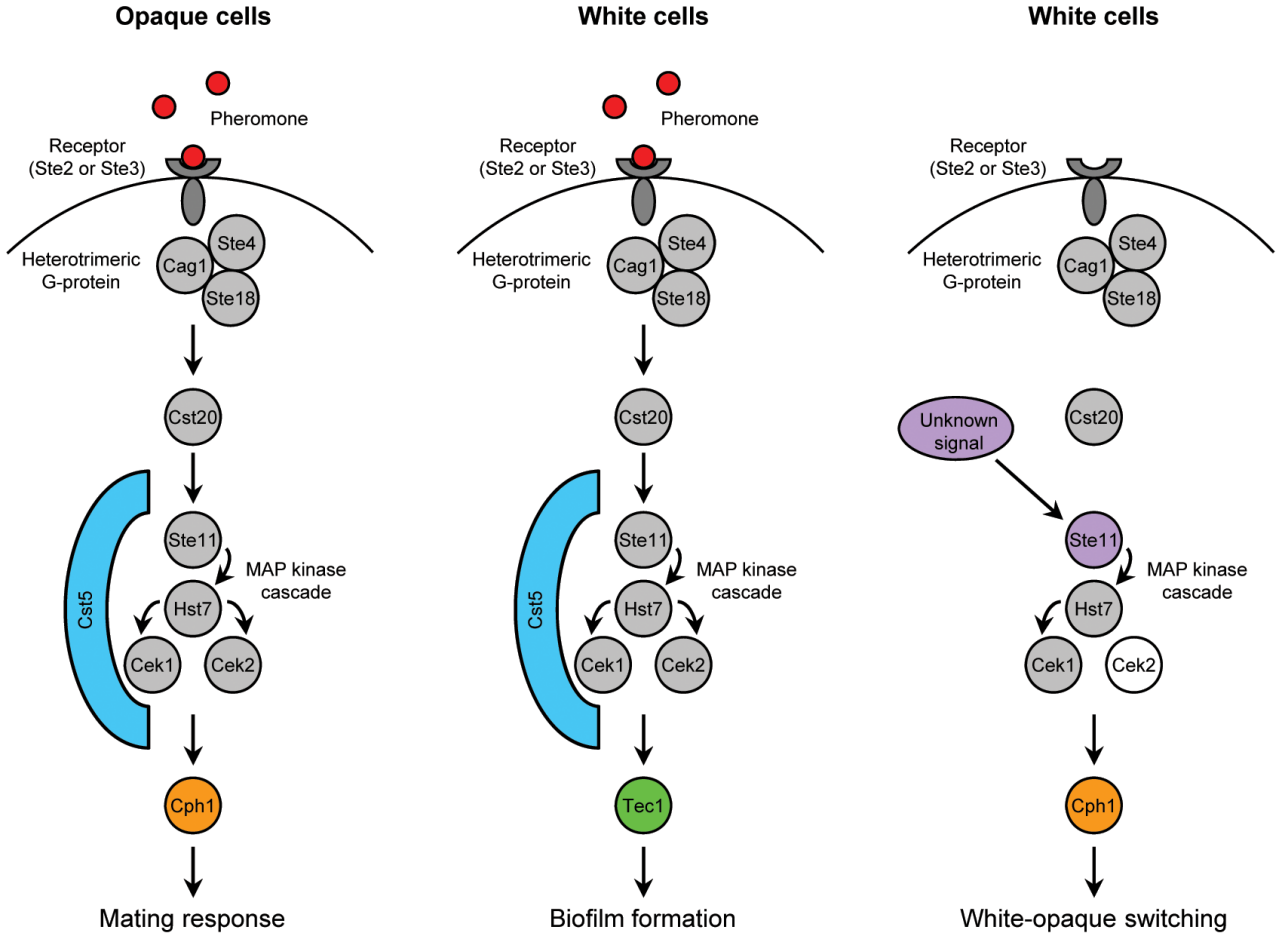
Schematic illustrating the different usages of the pheromone-responsive MAP kinase signaling pathway in white and opaque cells. As previously reported [22], [23], stimulation of the pathway by pheromone in opaque cells results in activation of the transcription factor Cph1 to induce the mating response (left), whereas white cells use the same pathway, but a different downstream transcription factor, Tec1, to promote biofilm formation in response to pheromone (middle). The scaffold protein Cst5 is required for both pheromone responses. Our present study shows that the MAP kinase pathway can also be activated in a different way in white cells, as by the hyperactive Ste11^ΔN467^ (right). This results in rewiring of the components, such that white cells now recruit the opaque-specific transcription factor Cph1 instead of Tec1 to promote a different behavioral response, switching to the opaque phase. Only one of the two MAP kinases, Cek1, is required for this response, while Cek2 is dispensable (indicated by the unfilled circle). In contrast to the pheromone-induced biofilm formation and mating response, Ste11-induced white-opaque switching does not depend on the scaffold protein Cst5. The signals that activate Ste11 in such an alternative way are unknown and may or may not involve the simultaneous presence of pheromone to induce white-opaque switching only under appropriate conditions. In our experiments, the upregulation of *CPH1* in white cells also induced biofilm formation in a Tec1-dependent manner, in line with two recent studies [51], [52] that also found a role for Cph1 in white cell biofilm formation (not shown in the figure).

White and opaque cells use the same upstream components of the pheromone-induced MAP kinase signaling pathway, but different transcription factors to effect their specific responses. White cells induce expression of *TEC1*, but not *CPH1*, whereas opaque cells induce expression of *CPH1*, but not *TEC1*, in response to pheromone [22], [23], [34]. It is not currently known how the upregulation of the alternative downstream transcription factor, *CPH1* in white cells and *TEC1* in opaque cells, in the presence of pheromone is blocked in the two cell types. In *Saccharomyces cerevisiae*, components of the pheromone-responsive MAP kinase cascade are also used for the induction of a different developmental program, invasive growth, under starvation conditions [36]. Here, the scaffold protein Ste5, which binds all three components of the MAP kinase cascade, acts as a conformational switch that gates the flow of information to ensure a proper physiological response to different inducing signals [37]. In *C. albicans*, the homologous scaffold protein Cst5 is required for both the Cph1-mediated mating response of opaque cells and Tec1-dependent biofilm formation of white cells [32]. The hyperactive Ste11^ΔN467^, which lacks the binding site for Cst5 [35], caused upregulation of *CPH1* instead of *TEC1* in white cells, *i.e.*, these cells had overcome the block in *CPH1* induction upon activation of the MAP kinase pathway (Fig. 11). However, the absence of Cst5 was not sufficient to enable wild-type Ste11 to induce switching of white cells to the opaque phase, indicating that additional signals are required for Ste11 to recruit Cph1 and promote switching. Overexpression of *CPH1* has been reported already a decade ago to increase mating efficiency [31]. At the time of that study, the linkage of mating to white-opaque switching had not yet been uncovered and the mating experiments were performed with white cells. While overexpression of *CPH1* may have enhanced the mating response of opaque cells that were already in the population, our results suggest that the increased mating efficiency might also have been caused by Cph1-induced switching of white cells to the opaque phase.

We observed that some of the kinases identified in our study efficiently stimulated white-opaque switching only when their expression was induced in the preculture, but not during growth on agar plates (*STE11* ^ΔN467^, *TPK1*), while others (*RAD53*, *TPK2*) promoted switching under both conditions (see supplemental Table S2). Indeed, growth conditions affected the inducibility of white-opaque switching by overexpressed *STE11* ^ΔN467^ and *CPH1*: Growth of the cells under static conditions in microtiter plates, a procedure that we used to streamline screening of the library, resulted in more efficient switching than the usual culturing in Erlenmeyer flasks or glass tubes with shaking. Under the latter conditions, the switching rates obtained after Tet-induced *CPH1* expression were only slightly reduced, but no significant induction of switching by Ste11^ΔN467^ was observed, indicating that white cells could more easily be stimulated to switch when growing on the bottom of a microtiter plate (data not shown). A reduced growth rate, which facilitates Wor1 accumulation in a cell [24], or local buildup of higher CO_2_ levels, which promote white-opaque switching [25], may contribute to this effect. In this regard, it is interesting to note that increased CO_2_ concentrations were reported to induce white-opaque switching only when the cells were grown on agar plates, but not in liquid culture [25], and GlcNAc induces switching only in aged, but not in fresh cultures [26]. In addition, Tet-induced expression of Flo8, another transcription factor that was recently found to be involved in the regulation of white-opaque switching, induced switching only in the presence of elevated CO_2_ concentrations [29]. Evidently, the ability of a regulatory factor to induce switching depends on the environmental conditions, because these will affect the activity of additional regulators.

It has been proposed that the pheromone-induced biofilm formation response observed in white cells has evolved *via* the recruitment of components from the ancestral pheromone response pathway (all upstream components, from the pheromone receptor to the MAP kinases Cek1 and Cek2) and the filamentation pathway (the transcription factor Tec1) as well as target genes for biofilm formation [22]. Our results demonstrate that this signaling pathway can be used in a highly flexible way, depending not only on the cell type but also on the manner in which it is activated. The downstream transcription factor Cph1 mediates the mating response of opaque cells, but can also stimulate white cells to switch to the opaque phase to become mating-competent. In the latter case, one of the two MAP kinases, Cek1, is specifically recruited to transmit the signal from activated Ste11 to Cph1, whereas Cek1 and Cek2 have at least partially redundant functions in the pheromone-induced biofilm formation of white cells and in the mating response of opaque cells [23], [31]. *CPH1* was only required for the induction of white-opaque switching by the artificially activated Ste11, but not for the induction of switching by the tested environmental conditions and for spontaneous switching, in agreement with findings by other researchers [23]. Of note, Cek1 is also known to be activated upon cell wall stress, for example after treatment with the cell wall disturbing agent tunicamycin, but Cph1 has not been implicated as a Cek1 downstream target under these conditions [38], [39], and we were unable to stimulate white-opaque switching with tunicamycin (unpublished results). In *MTL* heterozygous cells, the Cph1-dependent MAP kinase pathway induces filamentous growth under starvation conditions [40], [41]. Our results provide further evidence that certain transcription factors (Cph1, Czf1, Efg1, Flo8) are involved in the regulation of both filamentation and white-opaque switching.

It is conceivable that the N-terminally truncated, hyperactive form of Ste11, which induced *CPH1* upregulation in white cells and switching to the opaque phase, reflects a normal function of the MAP kinase pathway when activated by an unknown physiological signal. In this respect, it is remarkable that white-opaque switching, which was thought to be restricted to *MTL* homozygous strains, has recently been observed in *MTL* **a**/α strains under specific growth conditions [42]. So far, we have not found environmental conditions that promote white-opaque switching in a Cph1-dependent fashion and such conditions might be encountered only in suitable host niches *in vivo*. It is even possible that the presence of pheromone is a prerequisite, and that additional signals are required to overcome the block in *CPH1* upregulation that is seen upon pheromone treatment of white cells *in vitro*. This, in turn, raises the intriguing hypothesis that *C. albicans* white cells may have the ability to react to the presence of a potential mating partner (pheromone-secreting opaque cells) by switching to the opaque phase and thus become themselves mating-competent in an appropriate environment.

## Materials and Methods

### Strains and growth conditions

The *C. albicans* strains used in this study are listed in supplemental Table S3. All strains were stored as frozen stocks with 15% glycerol at −80°C. The strains were subcultured separately in the white and opaque phases at room temperature on agar plates containing Lee's medium, pH 6.8 [43], and 5 µg/ml phloxine B, which selectively stains opaque colonies pink [44]. Strains were routinely grown in YPD (10 g yeast extract, 20 g peptone, 20 g glucose) or SD (1.7 g yeast nitrogen base without amino acids [YNB; BIO 101, Vista, Calif.], 20 g glucose per liter) liquid medium at 30°C in a shaking incubator. To prepare solid media, 1.5% agar was added to the media before autoclaving. For selection of nourseothricin-resistant transformants, 200 µg/ml nourseothricin (Werner Bioagents, Jena, Germany) was added to YPD agar plates. To obtain nourseothricin-sensitive derivatives in which the *SAT1* flipper cassette was excised by FLP-mediated recombination, transformants were grown overnight in YCB-BSA-YE medium (23.4 g yeast carbon base, 4 g bovine serum albumin, 2 g yeast extract per liter, pH 4.0) without selective pressure to induce the *SAP2* promoter controlling *caFLP* expression. Alternatively, strains containing a *SAT1* flipper cassette in which the *caFLP* gene is expressed from the *MAL2* promoter (as in plasmids pOP4G4, pOP4R2, and pMTLΔ1) were grown overnight in YPM medium (10 g yeast extract, 20 g peptone, 20 g maltose per liter) instead of YCB-BSA-YE to induce the *MAL2* promoter. Appropriate dilutions were plated on YPD agar plates containing 10 µg/ml nourseothricin and grown for 2 days at 30°C. Nourseothricin-sensitive clones were identified by their small colony size and confirmed by restreaking on YPD plates containing 200 µg/ml nourseothricin as described previously [45]. YCB-BSA-YE medium was also used to induce gene expression from the *OPT3* promoter.

To induce gene expression from the Tet promoter, white cells of the strains containing the protein kinase library were grown for 24 h at 30°C in Lee's medium in 96-well microtiter plates in the absence or presence of 50 µg/ml doxycycline, diluted 10^−5^, and spread on Lee's agar plates with or without 50 µg/ml doxycycline. The plates were incubated for 7 days at room temperature and the number of white, opaque, and mixed white/opaque colonies was determined. Induction of white-opaque switching by environmental signals was performed as described in the legend to Fig. 9. Incubation under anaerobic conditions was performed in an anaerobic jar (Anaerocult, Merck KGaA, Darmstadt, Germany) that generates an oxygen-free milieu in a CO_2_ atmosphere (18% CO_2_) within one hour.

### Construction of the Tet-inducible protein kinase expression library

To generate a comprehensive library containing all known or putative protein kinases and kinase regulators of *C. albicans*, we searched the *Candida* genome database (CGD, http://www.candidagenome.org) for genes that were annotated with this function. These genes were amplified from genomic DNA of strain SC5314 by PCR with primers that introduced a SalI site in front of the start codon and a BglII site behind the stop codon (primer sequences are available upon request). For genes with internal SalI or BglII sites, primers containing compatible XhoI and/or BamHI sites were used. The PCR products were appropriately digested and cloned in place of the *GFP* reporter gene in the SalI/BglII-digested vector pNIM6 [28], which is identical to the originally developed Tet-On vector pNIM1 [46], except that it contains the *TEF3* transcription termination sequence instead of the *ACT1* terminator behind *GFP*. All cloned genes were completely sequenced to confirm their identity and to exclude PCR errors.

### Other plasmid constructions

For the deletion of *CEK1*, *CEK2*, *CPH1*, *CST5*, *STE11*, *TEC1*, and *WOR2*, the upstream and downstream regions of the genes were amplified as SacI-SacII and XhoI-ApaI fragments, respectively, and cloned on both sides of the *SAT1* flipper cassette in plasmid pSFS5, a derivative of plasmid pSFS2 in which the *caFLP* gene is placed under the control of the *SAP2* promoter instead of the *MAL2* promoter [47] to result in pCEK1M1, pCEK2M1, pCPH1M1, pCST5M1, pSTE11M1, pTEC1M1, and pWOR2M3, respectively. For complementation of the *cek1*Δ and *cph1*Δ mutants, the complete *CEK1* and *CPH1* coding regions and flanking sequences were cloned as SacI-SacII fragments and inserted in place of the upstream flanking region of pCEK1M1 and pCPH1M1, generating pCEK1K1 and pCPH1K1, respectively. The *CPH1* and *TEC1* coding regions were also amplified and inserted in plasmid pNIM6 to generate doxycycline-inducible expression cassettes, as described above for the protein kinase expression library. For deletion of the *MTL* **a** or *MTL*α locus of strain SC5314, ca. 0.8 kb of the common flanking regions were amplified as SacI-SacII and XhoI-ApaI fragments and cloned on both sides of the *SAT1* flipper cassette in plasmid pSFS2 to generate pMTLΔ1. To express *CPH1* from the *OPT3* promoter, the *CPH1* coding sequence was substituted for *GFP* in plasmid pOPT3G22 [33], resulting in pOPT3-CPH1. HA-tagged versions of *CEK1*, *CEK2*, and *CPH1* were generated by amplifying the C-terminal parts of the genes with primers that introduced a BamHI site (encoding a Gly-Ser linker) instead of the stop codons. The PCR products were digested with BamHI and at internal or introduced SacI sites, fused to a PCR-amplified BamHI-SacII fragment containing the 3×HA-T*_ACT1_* sequences from pZCF36H2 [48], and substituted for the upstream flanking sequences in the corresponding deletion cassettes, resulting in pCEK1H1, pCEK2H1, and pCPH1H1.

### C. albicans transformation

*C. albicans* strains were transformed by electroporation [45] with the gel-purified inserts from the plasmids described above. The cassettes from the Tet-inducible protein kinase expression library were separated from the plasmid backbone by digestion with SacII/ApaI or SacII/KpnI (in some cases a partial digest was required). Gene deletion and reinsertion cassettes were excised from the corresponding plasmids by SacI/ApaI digestion. For insertion of P*_OP4_-GFP* and P*_OP4_-RFP* reporter fusions into various strain backgrounds, the ApaI-SacI fragments from pOP4G2 (with the *caSAT1* marker alone) or from pOP4G4 and pOP4R2 (with the recyclable *SAT1* flipper cassette) [18] were used. The correct integration of each construct was confirmed by Southern hybridization using the flanking sequences as probes. In each case, two independent series of strains were generated and used for further analysis.

### Isolation of genomic DNA and Southern hybridization

Genomic DNA from *C. albicans* strains was isolated as described previously [45]. The DNA was digested with appropriate restriction enzymes, separated on a 1% agarose gel, transferred by vacuum blotting onto a nylon membrane, and fixed by UV crosslinking. Southern hybridization with enhanced chemiluminescence-labeled probes was performed with the Amersham ECL Direct Nucleic Acid Labelling and Detection System (GE Healthcare UK Limited, Little Chalfont Buckinghamshire, UK) according to the instructions of the manufacturer.

### Biofilm formation

Overnight cultures of *C. albicans* strains in SD medium were diluted to 10^7^ cells/ml in Lee's medium with or without 50 µg/ml doxycycline and 500 µl of these suspensions was added to each well of a 24-well polystyrene microtiter plate. After 24 h of incubation at 30°C, the wells were gently washed with PBS and imaged. Quantification of biofilm formation was performed as previously described [49], with some modifications. Three hundred microliters of the cell suspensions was added to each well of a 96-well polystyrene microtiter plate. A well containing Lee's medium without cells was included as reference. After 24 h of incubation at 30°C, the medium was aspirated and the wells were washed three times with 200 µl sterile PBS. Subsequently, 100 µl crystal violet solution (1%) was added to each well for 5 min. The wells were washed three times with sterile water and bound crystal violet was released by adding 200 µl of 33% acetic acid. The obtained solution was transferred to a new microtiter plate and the absorbance read at 595 nm.

### Reverse transcription-quantitative polymerase chain reaction (RT-qPCR)

Overnight cultures of *C. albicans* strains were diluted 10^−2^ in Lee's medium with 50 µg/ml doxycycline and grown for 18 h at 30°C in individual wells of a flat-bottomed 96-well polystyrene microtiter plate. Cells were harvested and total RNA was extracted by the hot acidic phenol method [50] combined with a purification step with the RNeasy mini kit (Qiagen, Hilden, Germany), and treated with Turbo DNA-free DNase (Ambion, Austin, TX). cDNA was synthesized using 500 ng of total RNA from each sample with the Superscript III Super Mix (Invitrogen, Karlsruhe, Germany). PCR was performed on a MyiQ Real-time PCR system using the iQ SYBR Green Supermix kit (Bio-Rad Laboratories, Hercules, CA). Relative mRNA levels, adjusted to *ACT1* mRNA levels, were calculated using expression levels in the wild-type strain WO-1 (set to 1) as a reference. The primers used are listed in supplemental Table S4.

### Detection of HA-tagged proteins by western immunoblotting

Overnight cultures of the strains were tenfold diluted in 10 ml fresh Lee's medium with and without doxycycline or α-pheromone (see below) and grown without shaking for 18 h at 30°C. The cells were pelleted by centrifugation, washed in 2.5 ml breaking buffer (100 mM Tris-HCl [pH 7.5], 200 mM NaCl, 20% glycerol, 5 mM EDTA), and resuspended in 500 µl breaking buffer supplemented with protease and phosphatase inhibitors (100 mM Tris-HCl [pH 7.5], 200 mM NaCl, 20% glycerol, 5 mM EDTA, 0.1% β-mercaptoethanol, cOmplete EDTA-free Protease Inhibitor Cocktail and PhosStop Phosphatase Inhibitor Cocktail [Roche Diagnostics GmbH, Mannheim, Germany]). An equal volume of 0.5-mm acid-washed beads was added to each tube. Cells were mechanically disrupted on a FastPrep-24 cell-homogenizer (MP Biomedicals, Solon, USA) for two 40-seconds intervals, with 1 min on ice between each cycle. Samples were centrifuged at 13,000 rpm for 10 min at 4°C, the supernatant was collected, and the protein concentration was quantified using the Bradford protein assay. Extracts were heated for 5 min at 95°C, and equal amounts of protein of each sample were separated on an SDS-10% polyacrylamide gel. Separated proteins were transferred onto a nitrocellulose membrane with a Mini-Protean System (Bio-Rad, Munich, Germany) and stained with Ponceau S to control for equal loading. Membranes were blocked in 5% milk in PBS at room temperature for 1 hour and subsequently incubated overnight at 4°C with rat monoclonal anti-HA-Peroxidase antibody (Roche Diagnostics GmbH, Mannheim, Germany). Membranes were washed in PBS containing 0.1% Tween-20 and signals detected with ECL chemiluminescence detection system (GE Healthcare Bio-Sciences GmbH, Munich, Germany). For phosphatase treatment, cell extracts were washed in Amicon-ultra 10 K columns (Millipore Corporation, Billerica, USA) with three volumes of breaking buffer without EDTA and phosphatase inhibitors, and subsequently washed with two volumes of phosphatase buffer supplemented with 1 mM MnCl_2_. Washed cell extracts were incubated with λ Protein Phosphatase (New England Biolabs, Ipswich, USA) at 30°C for 30 min.

### Pheromone treatment

To investigate whether white-opaque switching could be induced by pheromone, white cells of *MTL* **a**/Δ derivatives of strain SC5314 were grown overnight in SD medium at room temperature, washed with water, and resuspended in Lee's medium or Spider medium to a concentration of 10^7^ cells/ml. α-pheromone (Seqlab, Göttingen, Germany) was added at a concentration of 10 µg/ml, and the cultures were incubated at room temperature for 24 h. An appropriate dilution of the cultures was spread on Lee's agar plates with phloxin B and incubated for 7 days at room temperature to determine the frequency of opaque colonies. The experiment was repeated several times using additional additives and conditions along the pheromone treatment, including addition of 1 µg/ml pepstatin A (to prevent pheromone degradation by secreted aspartic proteases) and/or 10% FCS, incubation in the presence of 1% CO_2_, incubation at 30°C or 37°C, and elongation of the treatment time to 48 h. In addition, the cells were also grown in the absence or presence of α-pheromone on Lee's agar plates without or with 1.2 M sorbitol in normal air or in a 1% CO_2_ atmosphere.

To test the effect of **a**-pheromone on the *MTL*α/α strain WO-1, opaque cells of *MTL* **a**/Δ and *MTL*α/Δ derivatives of strain SC5314 were mixed (to serve as a source of both **a**- and α-pheromone) and incubated with white cells of nourseothricin-resistant derivatives of strain WO-1 (the wild-type strains WOP4G2A and -B and the *tec1*Δmutants WTEC1M3A and -B) to a final concentration of 10^7^ cells/ml in Lee's medium. The proportions of cells in the mixture were 50% white test cells, 25% opaque **a**-cells, and 25% opaque α-cells. The cell suspensions were incubated at room temperature for 24 h, diluted, and spread on Lee's agar plates with phloxin B and 100 µg/ml nourseothricin, on which only the nourseothricin-resistant test strains could grow. The plates were incubated for 7 days at room temperature and the presence of opaque colonies was recorded. In control experiments, we incubated *RFP*-expressing *MTL* **a**/Δ and *MTL*α/Δ opaque cells (strains SCMTLαM2BOP4R22 and SCMTL**a**M2BOP4R22) together with *GFP*-expressing opaque cells of strain WO-1 (strains WOP4G2A and -B). Microscopic inspection of the cells after 6 h of incubation showed that both *GFP*- and *RFP*-expressing cells formed shmoos, demonstrating that sufficient **a**-pheromone was produced by the mixture to induce the mating response in the *MTL*α/α strain WO-1.

## Supporting Information

Figure S1 Induction of white-opaque switching by Tet-induced expression of the protein kinases *MPS1*, *RAD53*, *TPK1*, and *TPK2* in the wild-type strain WO-1 and in *cph1*Δ (A) and *ste11*Δ mutants (B). White cells of the strains were grown for 18 h in liquid Lee's medium at 30°C in the presence of doxycycline to induce expression of the kinases, diluted, and plated on Lee's agar plates without doxycycline to determine the percentage of opaque (black bars) and mixed white/opaque colonies (gray bars). Two independently constructed strains were tested in each case; the untransformed parental strain WO-1 served as control in four sets of experiments. Results are the means and standard deviations from three biological replicates. Only background switching frequencies were observed in all strains when the precultures were grown in the absence of doxycycline (not shown).
(PDF)

Table S1 Tet-inducible *Candida albicans* protein kinase library.
(PDF)

Table S2 Results of the screening for protein kinases that induce white-opaque switching. Three white colonies of each strain were combined and suspended in SD medium, grown overnight at 30°C, and diluted 1∶10 in Lee's medium without or with 50 µg/ml doxycycline in 96-well microtiter plates. After 24 h of incubation at 30°C, the cells were thoroughly suspended, diluted 10^−5^, and 100 µl was spread on agar plates (Lee's medium with phloxine B) in the absence or presence of 50 µg/ml doxycycline. The number of white, opaque, and mixed white/opaque colonies was scored after 7 days of growth at room temperature. Two independent transformants were tested for each Tet-inducible protein kinase. The parental strain WO-1 and a strain expressing the transcription factor *CZF1* (a positive regulator of white-opaque switching) were included as negative and positive controls, respectively, in each set of experiments (results are from two representative experiments with these strains).
(XLSX)

Table S3 *C. albicans* strains used in this study. Derivatives expressing protein kinases or transcription factors from the Tet or *OPT3* promoters are not included in the table.
(PDF)

Table S4 Primers used for RT-qPCR.
(PDF)
