# Supplementary material for: Activation of the Cph1-Dependent MAP Kinase Signaling Pathway Induces White-Opaque Switching in Candida albicans
Source: PLoS Pathog. 2013 Oct 10;9(10):e1003696. doi: 10.1371/journal.ppat.1003696 (PMC3795047; doi:10.1371/journal.ppat.1003696)
Supplement: Figure S1 — Induction of white-opaque switching by Tet-induced expression of the protein kinases MPS1, RAD53, TPK1, and TPK2 in the wild-type strain WO-1 and in cph1Δ (A) and ste11Δ mutants (B). White cells of the strains were grown for 18 h in liquid Lee's medium at 30°C in the presence of doxycycline to induce expression of the kinases, diluted, and plated on Lee's agar plates without doxycycline to determine the percentage of opaque (black bars) and mixed white/opaque colonies (gray bars). Two independently constructed strains were tested in each case; the untransformed parental strain WO-1 served as control in four sets of experiments. Results are the means and standard deviations from three biological replicates. Only background switching frequencies were observed in all strains when the precultures were grown in the absence of doxycycline (not shown). (PDF) [file ppat.1003696.s001.pdf]

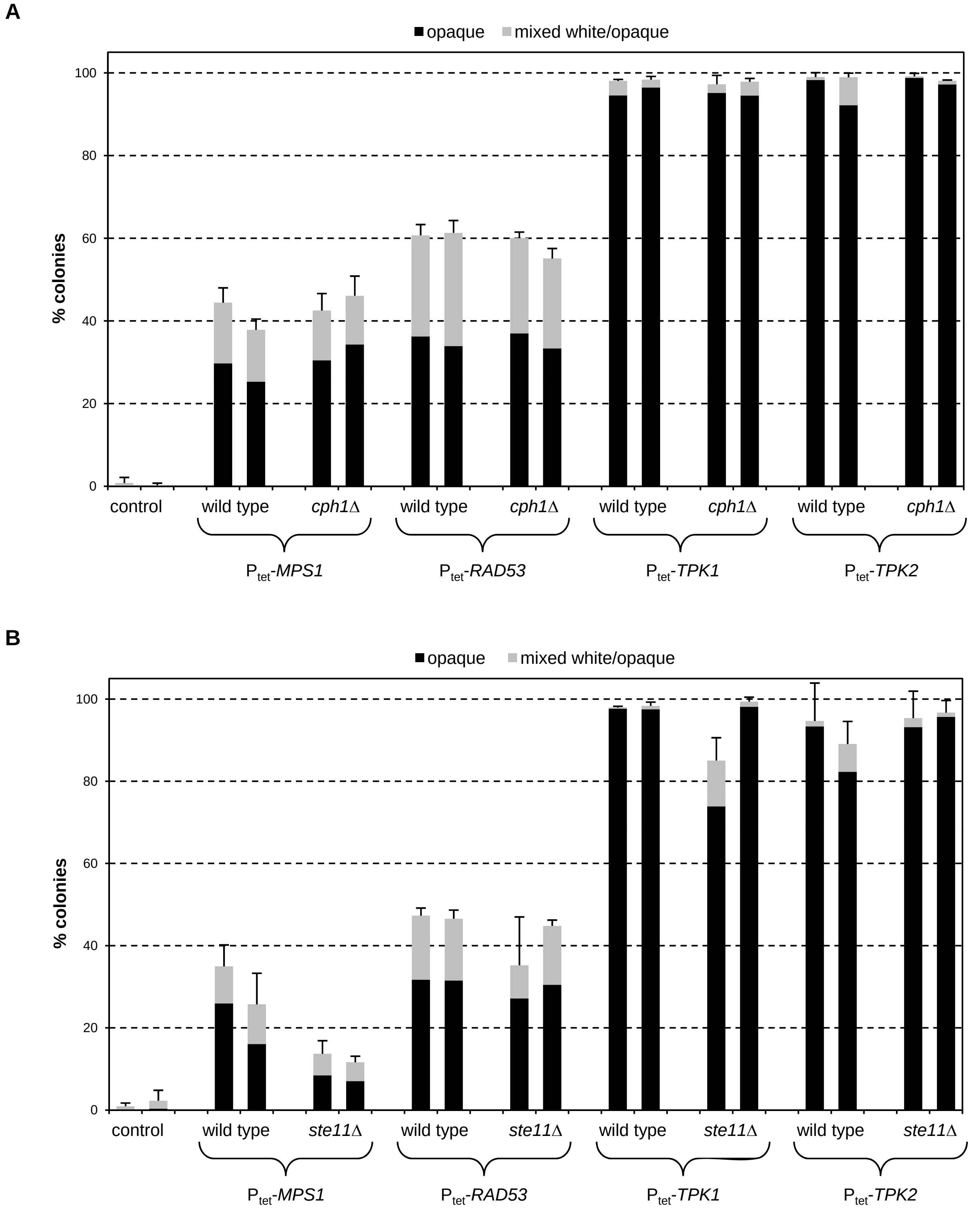

Fig. S1. Induction of white-opaque switching by Tet-induced expression of the protein kinases *MPS1*, *RAD53*, *TPK1*, and *TPK2* in the wild-type strain WO-1 and in *cph1*Δ (A) and *ste11*Δ mutants (B). White cells of the strains were grown for 18 h in liquid Lee's medium at 30°C in the presence of doxycycline to induce expression of the kinases, diluted, and plated on Lee's agar plates without doxycycline to determine the percentage of opaque (black bars) and mixed white/opaque colonies (gray bars). Two independently constructed strains were tested in each case; the untransformed parental strain WO-1 served as control in four sets of experiments. Results are the means and standard deviations from three biological replicates. Only background switching frequencies were observed in all strains when the precultures were grown in the absence of doxycycline (not shown).
