# Supplementary material for: Activation of the Cph1-Dependent MAP Kinase Signaling Pathway Induces White-Opaque Switching in Candida albicans
Source: PLoS Pathog. 2013 Oct 10;9(10):e1003696. doi: 10.1371/journal.ppat.1003696 (PMC3795047; doi:10.1371/journal.ppat.1003696)
Supplement: Table S1 — Tet-inducible Candida albicans protein kinase library. (PDF) [file ppat.1003696.s002.pdf]

Table S1: Tet-inducible *Candida albicans* protein kinase library

Library name	CGD name	orf 19 no.	Description/Annotation	Remarks [1]
AKL1	*AKL1*	5357	protein serine/threonine kinase activity (IEA)	
ATG1		3841	protein serine/threonine kinase activity (IEA)	
ATG1^{K87A}				dominant negative [1]
BCK1	*BCK1*	5162	MAP kinase kinase kinase activity (IEA)	
BCK1^{A1031P}				hyperactive [2]
BCY1	*BCY1*	2014	cAMP-dependent protein kinase regulator activity (IDA, IGI)	
BUB1	*BUB1*	2678	protein serine/threonine kinase activity (IEA)	
BUD32		4252	protein serine/threonine kinase activity (IEA), protein tyrosine kinase activity (IEA)	
BUR2	*BUR2*	4284	regulation of cyclin-dependent protein kinase activity (IEA)	
CAK1	*CAK1*	793	cyclin-dependent protein kinase activating kinase activity (IDA, IGI)	
CBK1	*CBK1*	4909	protein serine/threonine kinase activity (IGI, ISS)	
CBK1^{S546A}				dominant negative [3]
CCL1		4542	regulation of cyclin-dependent protein kinase activity (IEA)	
CCN1	*CCN1*	3207	cyclin-dependent protein kinase regulator activity (IGI, ISS)	
CCN1$^{\Delta C496}$				hyperactive [4]
CDC5	*CDC5*	6010	protein serine/threonine kinase activity (IEA)	
CDC7	*CDC7*	3561	protein serine/threonine kinase activity (IEA)	
CDC15		3545	protein serine/threonine kinase activity (IEA)	
CDC28	*CDC28*	3856	cyclin-dependent protein kinase activity (IDA, IGI, ISS)	
CDC28^{K37R}				dominant negative [5]
CDC37	*CDC37*	5531	regulation of stress-activated MAPK cascade (IEA)	
CEK1	*CEK1*	2886	protein kinase activity (ISS)	
CEK2	*CEK2*	460	MAP kinase activity (IGI, ISS)	
CHK1	*CHK1*	896	Histidine kinase	
CKA1	*CKA1*	7652	protein serine/threonine kinase activity (ISS)	
CKA2	*CKA2*	3530	protein serine/threonine kinase activity (ISS)	
CKB1	*CKB1*	2102	protein kinase regulator activity (IDA, ISS)	
CKB2	*CKB2*	4297	protein kinase regulator activity (IDA, ISS)	
CKS1	*CKS1*	1282	protein kinase activator activity (IEA)	
CLA4	*CLA4*	4890	protein serine/threonine kinase activity (IGI, ISS)	
CLA4$^{\Delta C891}$				dominant negative [6]
CLB2	*CLB2*	1446	cyclin-dependent protein kinase regulator activity (IDA, IGI, IMP, ISS)	
CLB4	*CLB4*	7186	cyclin-dependent protein kinase regulator activity (IMP, ISS)	
CLG1	*CLG1*	6146	regulation of cyclin-dependent protein kinase activity (IEA)	
CLN3	*CLN3*	1960	cyclin-dependent protein kinase regulator activity (IGI)	

CLN3$^{\Delta C332}$				hyperactive [4]
CMK1	*CMK1*	5911	protein serine/threonine kinase activity (IEA)	
CMK2		1754	calmodulin-dependent protein kinase activity (IEA)	
CRK1	*CRK1*	3523	MAP kinase activity (ISS), protein kinase activity (IDA)	
CSK1	*CSK1*	7208	MAP kinase activity (IDA, ISS)	
CST20	*CST20*	4242	protein kinase activity (IGI)	
CTK1		1619	cyclin-dependent protein kinase activity (IEA)	
CTK2		1041	*contributes_to* cyclin-dependent protein kinase activity (IEA)	
CTK3		5350	*S. cerevisiae* best hit is *CTK3*	
DBF2	*DBF2*	1223	protein serine/threonine kinase activity (ISA, EIA)	
DBF4	*DBF4*	5166	protein serine/threonine kinase activator activity (IEA)	
DUN1	*DUN1*	4002	protein serine/threonine kinase activity (IEA)	
FAR1	*FAR1*	7105	cyclin-dependent protein kinase inhibitor activity (IEA)	
FUN31	*FUN31*	7451	protein serine/threonine kinase activity (IEA)	
GCN2	*GCN2*	6913	eukaryotic translation initiation factor 2alpha kinase activity (IMP, ISS)	
GIN4	*GIN4*	663	protein serine/threonine kinase activity (IEA)	
HGC1	*HGC1*	6028	regulation of cyclin-dependent protein kinase activity (IMP)	
HGC1$^{\Delta C403}$				hyperactive [4]
HNT1	*HNT1*	2341	Protein with similarity to protein kinase C inhibitor-I	
HOG1	*HOG1*	895	MAP kinase activity (ISS), protein kinase activity (IDA)	
HOG1^{F321L}				hyperactive [7]
HRK1	*HRK1*	5408	protein serine/threonine kinase activity (IEA)	
HRR25		3476	protein serine/threonine kinase activity (IEA)	
HSL1	*HSL1*	4308	protein serine/threonine kinase activity (IEA)	
HSL1^{G74D}				dominant negative [8]
HST7	*HST7*	469	MAP kinase kinase activity (IGI, ISS), protein serine/threonine kinase activity (IEA)	
IKS1		428	protein serine/threonine kinase activity (IEA)	
IME2	*IME2*	2395	protein serine/threonine kinase activity (IEA)	
IPL1	*IPL1*	3474	protein serine/threonine kinase activity (IEA)	
IRE1	*IRE1*	5068	protein serine/threonine kinase activity (IEA)	
KIC1	*KIC1*	191	protein serine/threonine kinase activity (IEA)	
KIN2	*KIN2*	7510	protein serine/threonine kinase activity (IEA)	
KIN3	*KIN3*	5325	protein serine/threonine kinase activity (IEA)	
KIN4		3751	protein serine/threonine kinase activity (IEA)	
KIN28		6239	putative serine/threonine protein kinase	
KIS1	*KIS1*	4084	*contributes_to* AMP-activated protein kinase activity (IEA)	
KIS2	*KIS2*	4997	*S. cerevisiae* best hit is *SIP2*	
KNS1	*KNS1*	4979	protein serine/threonine kinase activity (IEA), protein tyrosine kinase activity (IEA)	
KSP1	*KSP1*	4432	protein serine/threonine kinase activity (IEA)	
MCK1		3459	protein serine/threonine/tyrosine kinase activity (IEA)	

MEC1	*MEC1*	1283	protein serine/threonine kinase activity (IEA)	
MEK1		1874	protein serine/threonine kinase activity (IEA)	
MKC1	*MKC1*	7523	MAP kinase activity (IGI, ISS)	
MKK2	*MKK2*	6889	protein serine/threonine kinase activity (IEA)	
MKK2$^{S317A\ T321A}$				dominant negative [9]
MOB2	*MOB2*	6044	protein kinase activator activity (IGI)	
MPS1	*MPS1*	7293	protein serine/threonine kinase activity (IEA)	
NIK1	*NIK1*	5181	protein histidine kinase activity (IDA, ISS)	
NPR1	*NPR1*	6232	protein serine/threonine kinase activity (IEA)	
PBS2	*PBS2*	7388	MAP kinase kinase activity (IMP, ISS), protein serine/threonine kinase activity (IEA)	
PBS2^{K233A}				dominant negative [10]
PCL1	*PCL1*	2649	regulation of cyclin-dependent protein kinase activity (IEA)	
PCL2	*PCL2*	403	cyclin-dependent protein kinase regulator activity (IEA)	
PCL5	*PCL5*	4012	cyclin-dependent protein kinase regulator activity (ISS)	
PCL5$^{T38A\ S43A}$				hyperactive (T.G., unpublished)
PCL7	*PCL7*	6225	regulation of cyclin-dependent protein kinase activity (IEA)	
PHO80		5755	regulation of cyclin-dependent protein kinase activity (IEA)	
PHO81	*PHO81*	7475	cyclin-dependent protein kinase inhibitor activity in *S. cerevisiae*	
PHO85	*PHO85*	6846	cyclin-dependent protein kinase activity (IGI, ISS)	
PKC1	*PKC1*	5901	protein kinase C activity (IDA, IGI, ISS)	
PKC1^{R399P}				hyperactive [11]
PKH1		5224	protein serine/threonine kinase activity (IEA)	
PKH3		1196	protein serine/threonine kinase activity (IEA)	
PKP1	*PDK2*	7281	putative pyruvate dehydrogenase kinase	
PRK1	*PRK1*	2605	protein serine/threonine kinase activity (IEA)	
PRR1		4347	receptor signaling protein serine/threonine kinase activity (IEA)	
PTK2	*PTK2*	3415	protein serine/threonine kinase activity (IEA)	
RAD53	*RAD53*	6936	protein serine/threonine/tyrosine kinase activity (IEA)	
RAD53^{D296A}				dominant negative [12]
RCK2	*RCK2*	2268	protein serine/threonine kinase activity (ISA, IEA)	
RIM11	*RIM11*	791	protein serine/threonine kinase activity (IEA)	
RIM15	*RIM15*	7044	protein serine/threonine kinase activity (IEA)	
RIO1		2320	protein serine/threonine kinase activity (IEA)	
RIO2	*RIO2*	6369	protein serine/threonine kinase activity (IEA)	
SAT4		3854	protein serine/threonine kinase activity (IEA)	
SCH9	*SCH9*	829	protein serine/threonine kinase activity (IEA)	
SHA3	*SHA3*	3669	protein serine/threonine kinase activity (IEA)	
SIP3		3047	protein kinase-related protein	
SKY1		2436	protein serine/threonine kinase activity (IEA)	
SLN1	*SLN1*	3256	protein histidine kinase activity (IDA, ISS)	

SNF1	*SNF1*	1936	protein serine/threonine kinase activity (IGI)	
SNF1$^{T208D\ \Delta C351}$				dominant negative [13]
SNF1^{D193A}				hyperactive [13]
SNF4	*SNF4*	5768	protein serine/threonine kinase activator activity (IEA)	
SOK1	*SOK1*	451	putative protein kinase	
SOL1	*SOL1*	6930	cyclin-dependent protein kinase inhibitor activity (IGI)	
SOL1$^{\Delta N106}$				hyperactive [14]
SPS1		3049	protein serine/threonine kinase activity (IEA)	
SSK2	*SSK2*	3775	MAP kinase kinase kinase activity (IEA)	
SSN3	*SSN3*	794	cyclin-dependent protein kinase activity (IEA)	
SSN8	*SSN8*	7355	cyclin-dependent protein kinase regulator activity (IEA)	
STE11	*STE11*	844	MAP kinase kinase kinase activity (IEA)	
STE11$^{\Delta N467}$				hyperactive [15]
STE50	*STE50*	1636	*S. cerevisiae* best hit is *STE50*	
SWE1	*SWE1*	4867	protein serine/threonine kinase activity (IEA), protein tyrosine kinase activity (IEA)	
TEL1	*TEL1*	5580	protein serine/threonine kinase activity (IEA)	
TOR1	*TOR1*	2290	protein serine/threonine kinase activity (IEA)	
TPK1	*TPK1*	4892	cAMP-dependent protein kinase activity (IDA, IMP, ISS)	
TPK2	*TPK2*	2277	cAMP-dependent protein kinase activity (IDA, IGI, IMP, ISS)	
VPS15		130	protein serine/threonine kinase activity (IEA)	
VPS34	*VPS34*	6243	protein kinase activity (IDA)	
YAK1	*YAK1*	147	protein serine/threonine kinase activity (IEA), protein tyrosine kinase activity (IEA)	
YCK2	*YCK2*	7001	protein serine/threonine kinase activity (IEA)	
YPK1		399	protein serine/threonine kinase activity (IEA)	
YPK1^{G534R}				dominant negative [16]
YPK1^{T705E}				hyperactive [16]
35		35	protein serine/threonine kinase activity (IEA)	
223		223	protein serine/threonine kinase activity (IEA)	
264		264	regulation of cyclin-dependent protein kinase activity (IEA)	
846		846	protein serine/threonine kinase activity (IEA)	
1341	*PRR2*	1341	protein serine/threonine kinase activity (IEA)	
2222		2222	protein serine/threonine kinase activity (IEA)	ORF is C-terminally truncated in CGD
2781		2781	protein serine/threonine kinase activity (IEA)	
3456		3456	protein serine/threonine kinase activity (IEA)	
3720		3720	*S. cerevisiae* best hit is *BCY1*	
3840		3840	protein serine/threonine kinase activity (IEA)	
4144		4144	transferase activity, transferring phosphorus-containing groups (IEA)	
4269		4269	protein kinase activity (IEA)	
4518		4518	protein serine/threonine kinase activity (IEA)	
4575		4575	transferase activity, transferring phosphorus-containing groups (IEA)	

5253		5253	protein serine/threonine kinase activity (IEA)	
5376		5376	protein serine/threonine kinase activity (IEA)	
6275		6275	protein serine/threonine kinase activity (IEA)	
6492		6492	protein serine/threonine kinase activity (IEA)	
7164		7164	protein serine/threonine kinase activity (IEA)	

[1] Potential hyperactive and dominant negative alleles of specific genes are highlighted in red and contain mutations that have this effect either in *C. albicans* or in homologous kinases of other organisms (references are given). ΔN and ΔC denote N- and C-terminal truncations, respectively. Alterations in the gene annotation in the current Assembly 21 in the *Candida* genome database are indicated.

References

1. Tekinay T, Wu MY, Otto GP, Anderson OR, Kessin RH (2006) Function of the *Dictyostelium discoideum* Atg1 kinase during autophagy and development. Eukaryot Cell 5: 1797-1806.
2. Lee KS, Levin DE (1992) Dominant mutations in a gene encoding a putative protein kinase (*BCK1*) bypass the requirement for a *Saccharomyces cerevisiae* protein kinase C homolog. Mol Cell Biol 12: 172-182.
3. He Y, Fang X, Emoto K, Jan YN, Adler PN (2005) The tricornered Ser/Thr protein kinase is regulated by phosphorylation and interacts with furry during *Drosophila* wing hair development. Mol Biol Cell 16: 689-700.
4. Tyers M, Tokiwa G, Nash R, Futcher B (1992) The Cln3-Cdc28 kinase complex of *S. cerevisiae* is regulated by proteolysis and phosphorylation. EMBO J 11: 1773-1784.
5. Leroy D, Birck C, Brambilla P, Samama JP, Ducommun B (1996) Characterisation of human cdc2 lysine 33 mutations expressed in the fission yeast *Schizosaccharomyces pombe*. FEBS Lett 379: 217-221.
6. Chiroli E, Fraschini R, Beretta A, Tonelli M, Lucchini G, et al. (2003) Budding yeast PAK kinases regulate mitotic exit by two different mechanisms. J Cell Biol 160: 857-874.
7. Bell M, Capone R, Pashtan I, Levitzki A, Engelberg D (2001) Isolation of hyperactive mutants of the MAPK p38/Hog1 that are independent of MAPK kinase activation. J Biol Chem 276: 25351-25358.
8. Calonge TM, O'Connell MJ (2006) Antagonism of Chk1 signaling in the G2 DNA damage checkpoint by dominant alleles of Cdr1. Genetics 174: 113-123.
9. Sun W, Kesavan K, Schaefer BC, Garrington TP, Ware M, et al. (2001) MEKK2 associates with the adapter protein Lad/RIBP and regulates the MEK5-BMK1/ERK5 pathway. J Biol Chem 276: 5093-5100.
10. Zanke BW, Rubie EA, Winnett E, Chan J, Randall S, et al. (1996) Mammalian mitogen-activated protein kinase pathways are regulated through formation of specific kinase-activator complexes. J Biol Chem 271: 29876-29881.
11. Nonaka H, Tanaka K, Hirano H, Fujiwara T, Kohno H, et al. (1995) A downstream target of *RHO1* small GTP-binding protein is *PKC1*, a homolog of protein kinase C, which leads to activation of the MAP kinase cascade in *Saccharomyces cerevisiae*. EMBO J 14: 5931-5938.
12. Chehab NH, Malikzay A, Appel M, Halazonetis TD (2000) Chk2/hCds1 functions as a DNA damage checkpoint in G_1 by stabilizing p53. Genes Dev 14: 278-288.
13. Woods A, Azzout-Marniche D, Foretz M, Stein SC, Lemarchand P, et al. (2000) Characterization of the role of AMP-activated protein kinase in the regulation of glucose-activated gene expression using constitutively active and dominant negative forms of the kinase. Mol Cell Biol 20: 6704-6711.
14. Atir-Lande A, Gildor T, Kornitzer D (2005) Role for the SCF^{CDC4} ubiquitin ligase in *Candida albicans* morphogenesis. Mol Biol Cell 16: 2772-2785.
15. Cairns BR, Ramer SW, Kornberg RD (1992) Order of action of components in the yeast pheromone response pathway revealed with a dominant allele of the STE11 kinase and the multiple phosphorylation of the STE7 kinase. Genes Dev 6: 1305-1318.
16. Tanoue D, Kobayashi T, Sun Y, Fujita T, Takematsu H, et al. (2005) The requirement for the hydrophobic motif phosphorylation of Ypk1 in yeast differs depending on the downstream events, including endocytosis, cell growth, and resistance to a sphingolipid biosynthesis inhibitor, ISP-1. Arch Biochem Biophys 437: 29-41.
