# Supplementary material for: Activation of the Cph1-Dependent MAP Kinase Signaling Pathway Induces White-Opaque Switching in Candida albicans
Source: PLoS Pathog. 2013 Oct 10;9(10):e1003696. doi: 10.1371/journal.ppat.1003696 (PMC3795047; doi:10.1371/journal.ppat.1003696)
Supplement: Table S3 — C. albicans strains used in this study. Derivatives expressing protein kinases or transcription factors from the Tet or OPT3 promoters are not included in the table. (PDF) [file ppat.1003696.s004.pdf]

Table S3. *C. albicans* strains used in this study

Strain [a]	Parent	Relevant genotype or characteristics [b]	Reference
WO-1		wild-type strain, *MTL*α/α	[1]
SC5314		wild-type strain, *MTL***a**/α	[2]
SCMTL**a**M1A and -B	SC5314	*mtl***a**Δ::*SAT1-FLIP*/*MTL*α	this study
SCMTL**a**M2A	SCMTL**a**M1A	*mtl***a**Δ::*FRT*/*MTL*α	this study
SCMTL**a**M2B	SCMTL**a**M1B	*mtl***a**Δ::*FRT*/*MTL*α	this study
SCMTLαM1A and -B	SC5314	*MTL***a**/*mtl*αΔ::*SAT1-FLIP*	this study
SCMTLαM2A	SCMTLαM1A	*MTL***a**/*mtl*αΔ::*FRT*	this study
SCMTLαM2B	SCMTLαM1B	*MTL***a**/*mtl*αΔ::*FRT*	this study
*czf1*Δ, *wor1*Δ, and *wor2*Δ mutants			
WCZF1M4A and -B	WO-1	*czf1*Δ::*FRT*/*czf1*Δ::*FRT*	[3]
WWOR1M6A and -B	WO-1	*wor1*Δ::*FRT*/*wor1*Δ::*FRT*/*wor1*Δ::*FRT*	[3]
WWOR2M1A and -B	WO-1	*WOR2*/*wor2*Δ::*SAT1-FLIP*	this study
WWOR2M2A	WWOR2M1A	*WOR2*/*wor2*Δ::*FRT*	this study
WWOR2M2B	WWOR2M1B	*WOR2*/*wor2*Δ::*FRT*	this study
WWOR2M3A	WWOR2M2A	*wor2*Δ::*FRT*/*wor2*Δ::*SAT1-FLIP*	this study
WWOR2M3B	WWOR2M2B	*wor2*Δ::*FRT*/*wor2*Δ::*SAT1-FLIP*	this study
WWOR2M4A	WWOR2M3A	*wor2*Δ::*FRT*/*wor2*Δ::*FRT*	this study
WWOR2M4B	WWOR2M3B	*wor2*Δ::*FRT*/*wor2*Δ::*FRT*	this study
*cek1*Δ mutants and complemented strains			
WCEK1M1A and -B	WO-1	*CEK1*/*cek1*Δ::*SAT1-FLIP*	this study
WCEK1M2A	WCEK1M1A	*CEK1*/*cek1*Δ::*FRT*	this study
WCEK1M2B	WCEK1M1B	*CEK1*/*cek1*Δ::*FRT*	this study
WCEK1M3A	WCEK1M2A	*cek1*Δ::*FRT*/*cek1*Δ::*SAT1-FLIP*	this study

WCEK1M3B	WCEK1M2B	*cek1*Δ::*FRT*/*cek1*Δ::*SAT1-FLIP*	this study
WCEK1M4A	WCEK1M3A	*cek1*Δ::*FRT*/*cek1*Δ::*FRT*	this study
WCEK1M4B	WCEK1M3B	*cek1*Δ::*FRT*/*cek1*Δ::*FRT*	this study
WCEK1MK1A	WCEK1M4A	*cek1*Δ::*FRT*/*CEK1-SAT1-FLIP*	this study
WCEK1MK1B	WCEK1M4B	*cek1*Δ::*FRT*/*CEK1-SAT1-FLIP*	this study
WCEK1MK2A	WCEK1MK1A	*cek1*Δ::*FRT*/*CEK1-FRT*	this study
WCEK1MK2B	WCEK1MK1B	*cek1*Δ::*FRT*/*CEK1-FRT*	this study
*cek2*Δ mutants			
WCEK2M1A and -B	WO-1	*CEK2*/*cek2*Δ::*SAT1-FLIP*	this study
WCEK2M2A	WCEK2M1A	*CEK2*/*cek2*Δ::*FRT*	this study
WCEK2M2B	WCEK2M1B	*CEK2*/*cek2*Δ::*FRT*	this study
WCEK2M3A	WCEK2M2A	*cek2*Δ::*FRT*/*cek2*Δ::*SAT1-FLIP*	this study
WCEK2M3B	WCEK2M2B	*cek2*Δ::*FRT*/*cek2*Δ::*SAT1-FLIP*	this study
WCEK2M4A	WCEK2M3A	*cek2*Δ::*FRT*/*cek2*Δ::*FRT*	this study
WCEK2M4B	WCEK2M3B	*cek2*Δ::*FRT*/*cek2*Δ::*FRT*	this study
*cek1*Δ *cek2*Δ double mutants			
WΔ*cek2*CEK1M1A	WCEK2M4A	*CEK1*/*cek1*Δ::*SAT1-FLIP* *cek2*Δ::*FRT*/*cek2*Δ::*FRT*	this study
WΔ*cek2*CEK1M1B	WCEK2M4B	*CEK1*/*cek1*Δ::*SAT1-FLIP* *cek2*Δ::*FRT*/*cek2*Δ::*FRT*	this study
WΔ*cek2*CEK1M2A	WΔ*cek1*CEK1M1A	*CEK1*/*cek1*Δ::*FRT* *cek2*Δ::*FRT*/*cek2*Δ::*FRT*	this study
WΔ*cek2*CEK1M2B	WΔ*cek1*CEK1M1B	*CEK1*/*cek1*Δ::*FRT* *cek2*Δ::*FRT*/*cek2*Δ::*FRT*	this study
WΔ*cek2*CEK1M3A	WΔ*cek1*CEK1M2A	*cek1*Δ::*FRT*/*cek1*Δ::*SAT1-FLIP* *cek2*Δ::*FRT*/*cek2*Δ::*FRT*	this study

WΔ*cek2*CEK1M3B	WΔ*cek1*CEK1M2B	*cek1*Δ::*FRT*/*cek1*Δ::*SAT1-FLIP* *cek2*Δ::*FRT*/*cek2*Δ::*FRT*	this study
WΔ*cek2*CEK1M4A	WΔ*cek1*CEK1M3A	*cek1*Δ::*FRT*/*cek1*Δ::*FRT* *cek2*Δ::*FRT*/*cek2*Δ::*FRT*	this study
WΔ*cek2*CEK1M4B	WΔ*cek1*CEK1M3B	*cek1*Δ::*FRT*/*cek1*Δ::*FRT* *cek2*Δ::*FRT*/*cek2*Δ::*FRT*	this study
*cph1*Δ mutants and complemented strains			
WCPH1M1A and -B	WO-1	*CPH1*/*CPH1*/*cph1*Δ::*SAT1-FLIP*	this study
WCPH1M2A	WCPH1M1A	*CPH1*/*CPH1*/*cph1*Δ::*FRT*	this study
WCPH1M2B	WCPH1M1B	*CPH1*/*CPH1*/*cph1*Δ::*FRT*	this study
WCPH1M3A	WCPH1M2A	*CPH1*/*cph1*Δ::*FRT*/*cph1*Δ::*SAT1-FLIP*	this study
WCPH1M3B	WCPH1M2B	*CPH1*/*cph1*Δ::*FRT*/*cph1*Δ::*SAT1-FLIP*	this study
WCPH1M4A	WCPH1M3A	*CPH1*/*cph1*Δ::*FRT*/*cph1*Δ::*FRT*	this study
WCPH1M4B	WCPH1M3B	*CPH1*/*cph1*Δ::*FRT*/*cph1*Δ::*FRT*	this study
WCPH1M5A	WCPH1M4A	*cph1*Δ::*FRT*/*cph1*Δ::*FRT*/*cph1*Δ::*SAT1-FLIP*	this study
WCPH1M5B	WCPH1M4B	*cph1*Δ::*FRT*/*cph1*Δ::*FRT*/*cph1*Δ::*SAT1-FLIP*	this study
WCPH1M6A	WCPH1M5A	*cph1*Δ::*FRT*/*cph1*Δ::*FRT*/*cph1*Δ::*FRT*	this study
WCPH1M6B	WCPH1M5B	*cph1*Δ::*FRT*/*cph1*Δ::*FRT*/*cph1*Δ::*FRT*	this study
WCPH1MK1A	WCPH1M6A	*cph1*Δ::*FRT*/*cph1*Δ::*FRT*/*CPH1-SAT1-FLIP*	this study
WCPH1MK1B	WCPH1M6B	*cph1*Δ::*FRT*/*cph1*Δ::*FRT*/*CPH1-SAT1-FLIP*	this study
WCPH1MK2A	WCPH1MK1A	*cph1*Δ::*FRT*/*cph1*Δ::*FRT*/*CPH1-FRT*	this study
WCPH1MK2B	WCPH1MK1B	*cph1*Δ::*FRT*/*cph1*Δ::*FRT*/*CPH1-FRT*	this study
*tec1*Δ mutants			
WTEC1M1A and -B	WO-1	*TEC1*/*tec1*Δ::*SAT1-FLIP*	this study
WTEC1M2A	WTEC1M1A	*TEC1*/*tec1*Δ::*FRT*	this study
WTEC1M2B	WTEC1M1B	*TEC1*/*tec1*Δ::*FRT*	this study

WTEC1M3A	WTEC1M2A	*tec1*Δ::*FRT*/*tec1*Δ::*SAT1-FLIP*	this study
WTEC1M3B	WTEC1M2B	*tec1*Δ::*FRT*/*tec1*Δ::*SAT1-FLIP*	this study
WTEC1M4A	WTEC1M3A	*tec1*Δ::*FRT*/*tec1*Δ::*FRT*	this study
WTEC1M4B	WTEC1M3B	*tec1*Δ::*FRT*/*tec1*Δ::*FRT*	this study
*ste11*Δ mutants			
WSTE11M1A and -B	WO-1	*STE11*/*ste11*Δ::*SAT1-FLIP*	this study
WSTE11M2A	WSTE11M1A	*STE11*/*ste11*Δ::*FRT*	this study
WSTE11M2B	WSTE11M1B	*STE11*/*ste11*Δ::*FRT*	this study
WSTE11M3A	WSTE11M2A	*ste11*Δ::*FRT*/*ste11*Δ::*SAT1-FLIP*	this study
WSTE11M3B	WSTE11M2B	*ste11*Δ::*FRT*/*ste11*Δ::*SAT1-FLIP*	this study
WSTE11M4A	WSTE11M3A	*ste11*Δ::*FRT*/*ste11*Δ::*FRT*	this study
WSTE11M4B	WSTE11M3B	*ste11*Δ::*FRT*/*ste11*Δ::*FRT*	this study
*cst5*Δ mutants			
WCST5M1A and -B	WO-1	*CST5*/*cst5*Δ::*SAT1-FLIP*	this study
WCST5M2A	WCST5M1A	*CST5*/*cst5*Δ::*FRT*	this study
WCST5M2B	WCST5M1B	*CST5*/*cst5*Δ::*FRT*	this study
WCST5M3A	WCST5M2A	*cst5*Δ::*FRT*/*cst5*Δ::*SAT1-FLIP*	this study
WCST5M3B	WCST5M2B	*cst5*Δ::*FRT*/*cst5*Δ::*SAT1-FLIP*	this study
WCST5M4A	WCST5M3A	*cst5*Δ::*FRT*/*cst5*Δ::*FRT*	this study
WCST5M4B	WCST5M3B	*cst5*Δ::*FRT*/*cst5*Δ::*FRT*	this study

Strains expressing 3xHA-tagged *CEK1*, *CEK2*, and *CPH1* in wild-type and mutant backgrounds			
WCEK1H11A and -B	WO-1	*CEK1/CEK1-HA-SAT1-FLIP*	this study
WCEK1H12A	WCEK1H11A	*CEK1/CEK1-HA-FRT*	this study
WCEK1H12B	WCEK1H11B	*CEK1/CEK1-HA-FRT*	this study
WΔ*cek2*CEK1H11A	WCEK2M4A	*CEK1/CEK1-HA-SAT1-FLIP* *cek2*Δ::*FRT*/*cek2*Δ::*FRT*	this study
WΔ*cek2*CEK1H11B	WCEK2M4B	*CEK1/CEK1-HA-SAT1-FLIP* *cek2*Δ::*FRT*/*cek2*Δ::*FRT*	this study
WΔ*cek2*CEK1H12A	WΔ*cek2*CEK1H11A	*CEK1/CEK1-HA-FRT* *cek2*Δ::*FRT*/*cek2*Δ::*FRT*	this study
WΔ*cek2*CEK1H12B	WΔ*cek2*CEK1H11B	*CEK1/CEK1-HA-FRT* *cek2*Δ::*FRT*/*cek2*Δ::*FRT*	this study
WCEK2H11A and -B	WO-1	*CEK2/CEK2-HA-SAT1-FLIP*	this study
WCEK2H12A	WCEK2H11A	*CEK2/CEK2-HA-FRT*	this study
WCEK2H12B	WCEK2H11B	*CEK2/CEK2-HA-FRT*	this study
WΔ*cek1*CEK2H11A	WCEK1M4A	*cek1*Δ::*FRT*/*cek1*Δ::*FRT* *CEK2/CEK2-HA-SAT1-FLIP*	this study
WΔ*cek1*CEK2H11B	WCEK1M4B	*cek1*Δ::*FRT*/*cek1*Δ::*FRT* *CEK2/CEK2-HA-SAT1-FLIP*	this study
WΔ*cek1*CEK2H12A	WΔ*cek1*CEK2H11A	*cek1*Δ::*FRT*/*cek1*Δ::*FRT* *CEK2/CEK2-HA-FRT*	this study
WΔ*cek1*CEK2H12B	WΔ*cek1*CEK2H11B	*cek1*Δ::*FRT*/*cek1*Δ::*FRT* *CEK2/CEK2-HA-FRT*	this study
WCPH1H11A and -B	WO-1	*CPH1/CPH1/CPH1-HA-SAT1-FLIP*	this study
WCPH1H12A	WCPH1H11A	*CPH1/CPH1/CPH1-HA-FRT*	this study
WCPH1H12B	WCPH1H11B	*CPH1/CPH1/CPH1-HA-FRT*	this study

WΔ*cek1*CPH1H11A	WCEK1M4A	*cek1*Δ::*FRT*/*cek1*Δ::*FRT* *CPH1*/*CPH1*/*CPH1-HA-SAT1-FLIP*	this study
WΔ*cek1*CPH1H11B	WCEK1M4B	*cek1*Δ::*FRT*/*cek1*Δ::*FRT* *CPH1*/*CPH1*/*CPH1-HA-SAT1-FLIP*	this study
WΔ*cek1*CPH1H12A	WΔ*cek1*CPH1H11A	*cek1*Δ::*FRT*/*cek1*Δ::*FRT* *CPH1*/*CPH1*/*CPH1-HA-FRT*	this study
WΔ*cek1*CPH1H12B	WΔ*cek1*CPH1H11B	*cek1*Δ::*FRT*/*cek1*Δ::*FRT* *CPH1*/*CPH1*/*CPH1-HA-FRT*	this study
WΔ*cek2*CPH1H11A	WCEK2M4A	*cek2*Δ::*FRT*/*cek2*Δ::*FRT* *CPH1*/*CPH1*/*CPH1-HA-SAT1-FLIP*	this study
WΔ*cek2*CPH1H11B	WCEK2M4B	*cek2*Δ::*FRT*/*cek2*Δ::*FRT* *CPH1*/*CPH1*/*CPH1-HA-SAT1-FLIP*	this study
WΔ*cek2*CPH1H12A	WΔ*cek2*CPH1H11A	*cek2*Δ::*FRT*/*cek2*Δ::*FRT* *CPH1*/*CPH1*/*CPH1-HA-FRT*	this study
WΔ*cek2*CPH1H12B	WΔ*cek2*CPH1H11B	*cek2*Δ::*FRT*/*cek2*Δ::*FRT* *CPH1*/*CPH1*/*CPH1-HA-FRT*	this study
WCPH1M4CPH1H11A	WCPH1M4A	*cph1*Δ::*FRT*/*cph1*Δ::*FRT*/*CPH1-HA-SAT1-FLIP*	this study
WCPH1M4CPH1H11B	WCPH1M4B	*cph1*Δ::*FRT*/*cph1*Δ::*FRT*/*CPH1-HA-SAT1-FLIP*	this study
WCPH1M4CPH1H12A	WCPH1M4CPH1H11A	*cph1*Δ::*FRT*/*cph1*Δ::*FRT*/*CPH1-HA-FRT*	this study
WCPH1M4CPH1H12B	WCPH1M4CPH1H11B	*cph1*Δ::*FRT*/*cph1*Δ::*FRT*/*CPH1-HA-FRT*	this study
SCΔ*mtl*αCPH1H11A	SCMTLαM2A	*CPH1-1-HA-SAT1-FLIP*/*CPH1*	this study
SCΔ*mtl*αCPH1H11B	SCMTLαM2B	*CPH1-1*/*CPH1-2-HA-SAT1-FLIP*	this study
SCΔ*mtl*αCPH1H12A	SCΔ*mtl*αCPH1H11A	*CPH1-1-HA-FRT*/*CPH1*	this study
SCΔ*mtl*αCPH1H12B	SCΔ*mtl*αCPH1H11B	*CPH1-1*/*CPH1-2-HA-FRT*	this study

Strains expressing P$_{OP4}$-*GFP* or P$_{OP4}$-*RFP* reporter fusions in wild-type and mutant backgrounds			
WOP4G2A and -B	WO-1	*OP4*/*OP4*/*op4*::P$_{OP4}$-*GFP-caSAT1*	this study
WOP4G42A [c]	WO-1	*OP4*/*OP4*/*op4*::P$_{OP4}$-*GFP-FRT*	[4]
WOP4R22A [c]	WO-1	*OP4*/*OP4*/*op4*::P$_{OP4}$-*RFP-FRT*	[4]
WCZF1M4AOP4G41	WCZF1M4A	*czf1*Δ::*FRT*/*czf1*Δ::*FRT* *OP4*/*OP4*/*op4*::P$_{OP4}$-*GFP-SAT1-FLIP*	this study
WCZF1M4AOP4G42 [c]	WCZF1M4AOP4G41	*czf1*Δ::*FRT*/*czf1*Δ::*FRT* *OP4*/*OP4*/*op4*::P$_{OP4}$-*GFP-FRT*	this study
WCZF1M4BOP4R21	WCZF1M4B	*czf1*Δ::*FRT*/*czf1*Δ::*FRT* *OP4*/*OP4*/*op4*::P$_{OP4}$-*RFP-SAT1-FLIP*	this study
WCZF1M4BOP4R22 [c]	WCZF1M4BOP4R21	*czf1*Δ::*FRT*/*czf1*Δ::*FRT* *OP4*/*OP4*/*op4*::P$_{OP4}$-*RFP-FRT*	this study
WWOR1M6AOP4G41	WWOR1M6A	*wor1*Δ::*FRT*/*wor1*Δ::*FRT*/*wor1*Δ::*FRT* *OP4*/*OP4*/*op4*::P$_{OP4}$-*GFP-SAT1-FLIP*	this study
WWOR1M6AOP4G42 [c]	WWOR1M6AOP4G41	*wor1*Δ::*FRT*/*wor1*Δ::*FRT*/*wor1*Δ::*FRT* *OP4*/*OP4*/*op4*::P$_{OP4}$-*GFP-FRT*	this study
WWOR1M6BOP4R21	WWOR1M6B	*wor1*Δ::*FRT*/*wor1*Δ::*FRT*/*wor1*Δ::*FRT* *OP4*/*OP4*/*op4*::P$_{OP4}$-*RFP-SAT1-FLIP*	this study
WWOR1M6BOP4R22 [c]	WWOR1M6BOP4R21	*wor1*Δ::*FRT*/*wor1*Δ::*FRT*/*wor1*Δ::*FRT* *OP4*/*OP4*/*op4*::P$_{OP4}$-*RFP-FRT*	this study
WWOR2M4AOP4G41	WWOR2M4A	*wor2*Δ::*FRT*/*wor2*Δ::*FRT* *OP4*/*OP4*/*op4*::P$_{OP4}$-*GFP-SAT1-FLIP*	this study
WWOR2M4AOP4G42 [c]	WWOR2M4AOP4G41	*wor2*Δ::*FRT*/*wor2*Δ::*FRT* *OP4*/*OP4*/*op4*::P$_{OP4}$-*GFP-FRT*	this study
WWOR2M4BOP4R21	WWOR2M4B	*wor2*Δ::*FRT*/*wor2*Δ::*FRT* *OP4*/*OP4*/*op4*::P$_{OP4}$-*RFP-SAT1-FLIP*	this study
WWOR2M4BOP4R22 [c]	WWOR2M4BOP4R21	*wor2*Δ::*FRT*/*wor2*Δ::*FRT* *OP4*/*OP4*/*op4*::P$_{OP4}$-*RFP-FRT*	this study

SCMTL**a**M2BOP4R21	SCMTL**a**M2B	*mtl***a**Δ::*FRT*/*MTL*α<br>*OP4*/*OP4*/*op4*::P_{OP4}-*RFP-SAT1-FLIP*	this study
SCMTL**a**M2BOP4R22	SCMTL**a**M2BOP4R21	*mtl***a**Δ::*FRT*/*MTL*α<br>*OP4*/*OP4*/*op4*::P_{OP4}-*RFP-FRT*	this study
SCMTLαM2BOP4R21	SCMTLαM2B	*MTL***a**/*mtl*αΔ::*FRT*<br>*OP4*/*OP4*/*op4*::P_{OP4}-*RFP-SAT1-FLIP*	this study
SCMTLαM2BOP4R22	SCMTLαM2BOP4R21	*MTL***a**/*mtl*αΔ::*FRT*<br>*OP4*/*OP4*/*op4*::P_{OP4}-*RFP-FRT*	this study

[a] Derivatives expressing protein kinases or transcription factors from the Tet or *OPT3* promoters are not included in the table.

[b] *SAT1-FLIP* denotes the *SAT1* flipper cassette; *FRT* is the FLP recombination target sequence. *CPH1*, *OP4*, and *WOR1* and are located on chromosome 1, which is trisomic in some stocks of strain WO-1, including the one that was used in this and a previous study [3]. The *CPH1* alleles of strain SC5314 were distinguished by the presence or absence of an internal EcoRV restriction site and arbitrarily designated *CPH1-1* and *CPH1-2*, respectively.

[c] P_{OP4}-*GFP* and P_{OP4}-*RFP* reporter strains that were used for Tet-induced expression of *CPH1* and *STE11*$^{\Delta N467}$.

References

1. Slutsky B, Staebell M, Anderson J, Risen L, Pfaller M, et al. (1987) "White-opaque transition": a second high-frequency switching system in *Candida albicans*. J Bacteriol 169: 189-197.

2. Gillum AM, Tsay EY, Kirsch DR (1984) Isolation of the *Candida albicans* gene for orotidine-5'-phosphate decarboxylase by complementation of *S. cerevisiae ura3* and *E. coli pyrF* mutations. Mol Gen Genet 198: 179-182.

3. Ramírez-Zavala B, Reuß O, Park Y-N, Ohlsen K, Morschhäuser J (2008) Environmental induction of white-opaque switching in *Candida albicans*. PLoS Pathog 4: e1000089.

4. Sasse C, Hasenberg M, Weyler M, Gunzer M, Morschhäuser J (2013) White-opaque switching of *Candida albicans* allows immune evasion in an environment-dependent fashion. Eukaryot Cell 12: 50-58.
