# Supplementary material for: Activation of the Cph1-Dependent MAP Kinase Signaling Pathway Induces White-Opaque Switching in Candida albicans
Source: PLoS Pathog. 2013 Oct 10;9(10):e1003696. doi: 10.1371/journal.ppat.1003696 (PMC3795047; doi:10.1371/journal.ppat.1003696)
Supplement: Table S4 — Primers used for RT-qPCR. (PDF) [file ppat.1003696.s005.pdf]

Table S4. Primers used for RT-qPCR

Primer	Sequence
ACT1RT	5`-AGTGTGACATGGATGTTAGAAAAGAATTATACGG-3`
ACT2RT	5`-ACAGAGTATTTTCTTTCTGGTGGAGCA-3`
CPH1LRT	5`-TATGACGCTTCTGGGTTTCC-3`
CPH1RRT	5`-ATCCCATGGCAATTTGTTGT-3`
TEC1LRT	5`-ACACTTGCAACCACACCAAA-3`
TEC1RRT	5`-TGCGTGTAGGAAACACCAAA-3`
WOR1LRT	5`-CCACCAGCAGTCAGTACCAA-3`
WOR1RRT	5`-TAGTCATTGGCATGGGTTCA-3`
